# Hepatoid adenocarcinoma of the lung with normal serum alpha-fetoprotein managed with thoracic radiotherapy followed by tislelizumab plus lenvatinib: a case report and systematic review

**DOI:** 10.3389/fonc.2026.1963662

**Published:** 2026-09-18

**Authors:** Jing Feng, Yongsheng Wang, Fan Li, Yanmei Wen, Wenhao Xu, Xiaobo Hu

**Affiliations:** 1 Department of Respiratory and Critical Care Medicine, West China School of Medicine, Sichuan University, Sichuan University Affiliated Chengdu Second People’s Hospital, Chengdu Second People’s Hospital, Chengdu, Sichuan, China; 2 Department of Pathology, West China School of Medicine, Sichuan University, Sichuan University Affiliated Chengdu Second People’s Hospital, Chengdu Second People’s Hospital, Chengdu, Sichuan, China

**Keywords:** alpha-fetoprotein, case report, hepatoid adenocarcinoma of the lung, systematic review, treatment

## Abstract

**Background:**

Hepatoid adenocarcinoma of the lung (HAL) is a rare pulmonary malignancy. This study presents a case of HAL with normal serum alpha-fetoprotein (AFP) and systematically reviews previously reported cases.

**Methods:**

The clinicopathologic and molecular characteristics, treatment, and outcome of the index case were described. For the systematic review, PubMed, Embase, Web of Science, China National Knowledge Infrastructure (CNKI), and Wanfang Data were searched from January 2000 to 4 July 2026. Patient-level data were extracted from eligible reports. Methodological quality was assessed using the Joanna Briggs Institute checklists. Overall survival (OS) was evaluated using Kaplan–Meier analysis and Cox regression.

**Results:**

The index patient was diagnosed with HAL, with normal serum AFP and no established actionable driver alterations identified. After thoracic radiotherapy followed by tislelizumab plus lenvatinib, the patient achieved a partial response and remained progression-free for nearly 10 months. The systematic review included 91 studies comprising 109 patients. The median age was 63 years. Men accounted for 91% of patients, and 86% of patients with available data had a smoking history. Stage III–IV disease was reported in 88/106 patients (83%). Serum AFP was elevated in 49/81 patients (60%), thyroid transcription factor 1 (TTF-1) was negative in 56/87 patients (64%), and genomic alterations were identified in 19/53 molecularly tested patients (36%). The median OS among 83 patients with available survival data was 14 months (95% CI, 9–19 months). Stage IV disease was associated with shorter OS in multivariable analysis (HR, 2.47; 95% CI, 1.18–5.19; P = 0.017). These findings should be interpreted in light of potential publication, selection, and reporting biases.

**Conclusion:**

HAL was most frequently reported in older men with a history of smoking. Most patients had stage III–IV disease at diagnosis, and OS was generally short. Clinical stage was associated with OS, whereas the prognostic value of serum AFP remained uncertain. No standard treatment has been established.

**Systematic review registration:**

https://www.crd.york.ac.uk/PROSPERO/, identifier CRD420261440722.

## Introduction

Hepatoid adenocarcinoma of the lung (HAL) is a rare pulmonary malignancy characterized by hepatocellular differentiation and was first described by Ishikura et al. in 1990 (1). HAL is generally associated with an aggressive clinical course and poor prognosis. Reported 1-, 3-, and 5-year overall survival (OS) rates are 40.0%, 35.0%, and 8%–19%, respectively, with a median OS of 14–18 months (2, 3). Elevated serum alpha-fetoprotein (AFP) levels have been reported in most patients with HAL, although serum AFP may remain normal in some cases. No standardized treatment strategy has been established for advanced HAL (4).

We report a case of HAL with normal serum AFP. The patient presented with dyspnea caused by airway compression from a mediastinal mass. Positron emission tomography/computed tomography (PET/CT) showed hypermetabolic thoracic lesions and a mildly hypermetabolic right renal mass. Bronchoscopic sampling established a diagnosis of hepatoid adenocarcinoma, and the combined pathologic, radiologic, and next-generation sequencing (NGS) findings favored primary HAL. After thoracic radiotherapy for airway compression, the patient received tislelizumab plus lenvatinib. Subsequent follow-up showed a partial response, accompanied by normalization of carcinoembryonic antigen (CEA) levels. The patient has remained progression-free for nearly 10 months. We also performed a systematic review of published HAL cases to summarize their clinicopathologic features, molecular profiles, treatment strategies, and clinical outcomes.

## Case presentation

A 72-year-old man with a 40-pack-year smoking history presented on August 24, 2025, with a two-month history of cough, wheezing, and progressive dyspnea. He had hypertension and no family history of malignancy. Physical examination revealed mild perioral cyanosis, diminished breath sounds over the left lower lung field, and bilateral wheezes and crackles. Arterial blood gas analysis showed hypoxemia (PaO_2_, 58 mmHg). Laboratory tests showed elevated CEA (36.2 ng/mL) and squamous cell carcinoma antigen (SCCA, 34.8 ng/mL) levels, whereas AFP (2.89 ng/mL) was within the normal range.

Chest CT revealed multiple soft-tissue-density nodules and masses in the mediastinum. Subsequent PET/CT showed hypermetabolic right paratracheal and subcarinal lymphadenopathy. The largest nodal lesion measured 4.6 × 3.7 × 2.7 cm and showed internal necrosis with intense radiotracer uptake (SUVmax, 26.4). These lesions compressed and narrowed the adjacent trachea, bronchi, and esophagus. A 3-mm soft-tissue nodule was also identified in the left main bronchus, and a mildly hypermetabolic mass was noted at the lower pole of the right kidney (SUVmax, 3.0). Contrast-enhanced abdominal CT and PET/CT identified no evidence of a hepatic or gastrointestinal primary tumor. Gastrointestinal endoscopy was not performed because of airway stenosis and patient refusal.

Bronchoscopy showed mucosal elevation and thickening in the left main bronchus, with a small polypoid nodule and marked stenosis of the left main bronchial orifice. Biopsy of the bronchial lesion was performed, and endobronchial ultrasound-guided transbronchial needle aspiration (EBUS-TBNA) was conducted at lymph node stations 4R and 7. The left main bronchial biopsy and EBUS-TBNA specimens from stations 4R and 7 showed concordant features of poorly differentiated carcinoma with hepatoid morphology. Immunohistochemistry (IHC) showed CK7 (+), Hepatocyte (+), SMARCA4/BRG1 retained, INI-1 retained, Ki-67 approximately 40%, CK20 (−), TTF-1 (−), Napsin A (−), NKX3.1 (−), PAX-8 (−), S100 (−), WT1 (−), and p40 (−). Subsequent pathology consultation confirmed these immunophenotypic findings and additionally showed AFP (−), CDX2 (−), and GATA3 (−). These findings supported a diagnosis of poorly differentiated hepatoid adenocarcinoma without a site-specific immunophenotype (**Figure 1****).**

**Figure 1:**
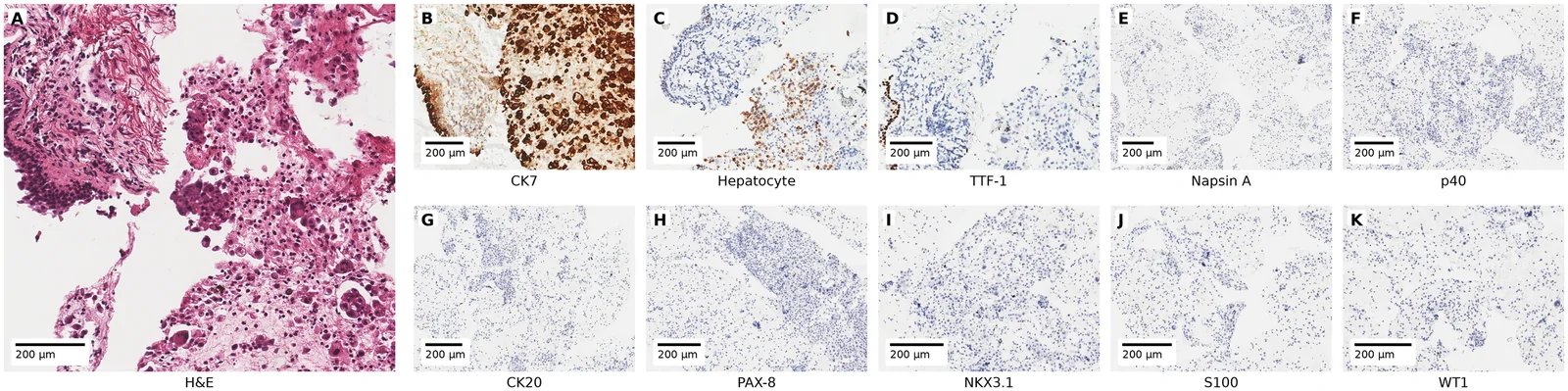
Histopathological and IHC findings of the left main bronchial biopsy specimen. **(A)** Hematoxylin and eosin (H&E) staining shows poorly differentiated carcinoma; **(B)** IHC staining for CK7 is positive in tumor cells; **(C)** IHC staining for Hepatocyte is positive in tumor cells; **(D)** IHC staining for TTF-1 is negative in tumor cells; **(E)** IHC staining for Napsin A is negative in tumor cells; **(F)** IHC staining for p40 is negative in tumor cells; **(G)** IHC staining for CK20 is negative in tumor cells; **(H)** IHC staining for PAX-8 is negative in tumor cells; **(I)** IHC staining for NKX3.1 is negative in tumor cells; **(J)** IHC staining for S100 is negative in tumor cells; and **(K)** IHC staining for WT1 is negative in tumor cells. Scale bars = 200 μm.

Targeted NGS covering 437 genes identified no established actionable driver alterations in *EGFR*, *ALK*, *ROS1*, *BRAF*, *MET*, *KRAS*, or *RET*. Notable alterations included *NKX2–1* p.V290M (13.11%), *TP53* p.R248Q (13.76%), *B2M* p.E97* (21.13%), *KEAP1* p.I125Mfs*24 *(22.19%), YAP1* p.R349H *(2.95%)*, and *CDK12* p.Q695* (11.54%). Tumor mutational burden (TMB) was 34 mutations/Mb, and the programmed death-ligand 1 (PD-L1) tumor proportion score (TPS) was 1%–5%.

Multidisciplinary review focused on determining the primary site of this poorly differentiated carcinoma with hepatoid differentiation. Given the absence of chronic liver disease and hepatic, gastrointestinal, or gonadal primary tumors on imaging, together with normal serum AFP and β-hCG levels and negative CK20 and CDX2 expression, metastatic hepatocellular carcinoma, gastrointestinal hepatoid adenocarcinoma, and germ-cell tumor were not supported. Urologic consultation favored a primary clear cell renal cell carcinoma for the right renal mass, and PAX-8 (−) did not support renal origin of the thoracic disease. The morphologic and IHC findings supporting hepatoid differentiation were not typical of conventional poorly differentiated non-small cell lung cancer (NSCLC). Based on the overall clinicopathologic and radiologic findings, primary HAL was favored (cT2aN3M0, stage IIIB, AJCC 9th edition).

With worsening airway compression, airway stent implantation was recommended but declined. Thoracic intensity-modulated radiotherapy (IMRT) was subsequently administered in two sequential courses: 15 Gy in five fractions to the mediastinal nodal lesions and left hilar lesion beginning on September 12, 2025, followed by 15 Gy in five fractions to the mediastinal nodal lesions beginning on September 18, 2025. Given the lack of an established standard systemic treatment for HAL, the treatment strategy was individualized after multidisciplinary discussion, taking into account the patient’s poor general condition and refusal of chemotherapy, the absence of actionable driver alterations, and the high TMB. Previous reports of clinical activity with immunotherapy and antiangiogenic therapy in HAL also informed the treatment decision (4). Tislelizumab (200 mg every 3 weeks) was therefore initiated on September 28, 2025, and lenvatinib (8 mg once daily) was added approximately 2 weeks later. Follow-up CT showed a partial response (**Figure 2**), accompanied by normalization of CEA levels. The thoracic lesions showing regression were within the radiation field. No measurable HAL lesion outside the radiation field was available for independent response assessment. By July 20, 2026, the patient had received 15 cycles of tislelizumab and had remained on lenvatinib for approximately 9 months. During treatment, a transient grade 2 elevation in serum transaminases was observed and improved after hepatoprotective treatment without requiring treatment interruption or dose reduction. Treatment with both agents was ongoing at the last follow-up, with the patient remaining progression-free for nearly 10 months after initiation of tislelizumab. The right renal lesion showed no significant radiologic change during follow-up. The clinical course and treatment timeline are summarized in **Table 1**.

**Figure 2:**
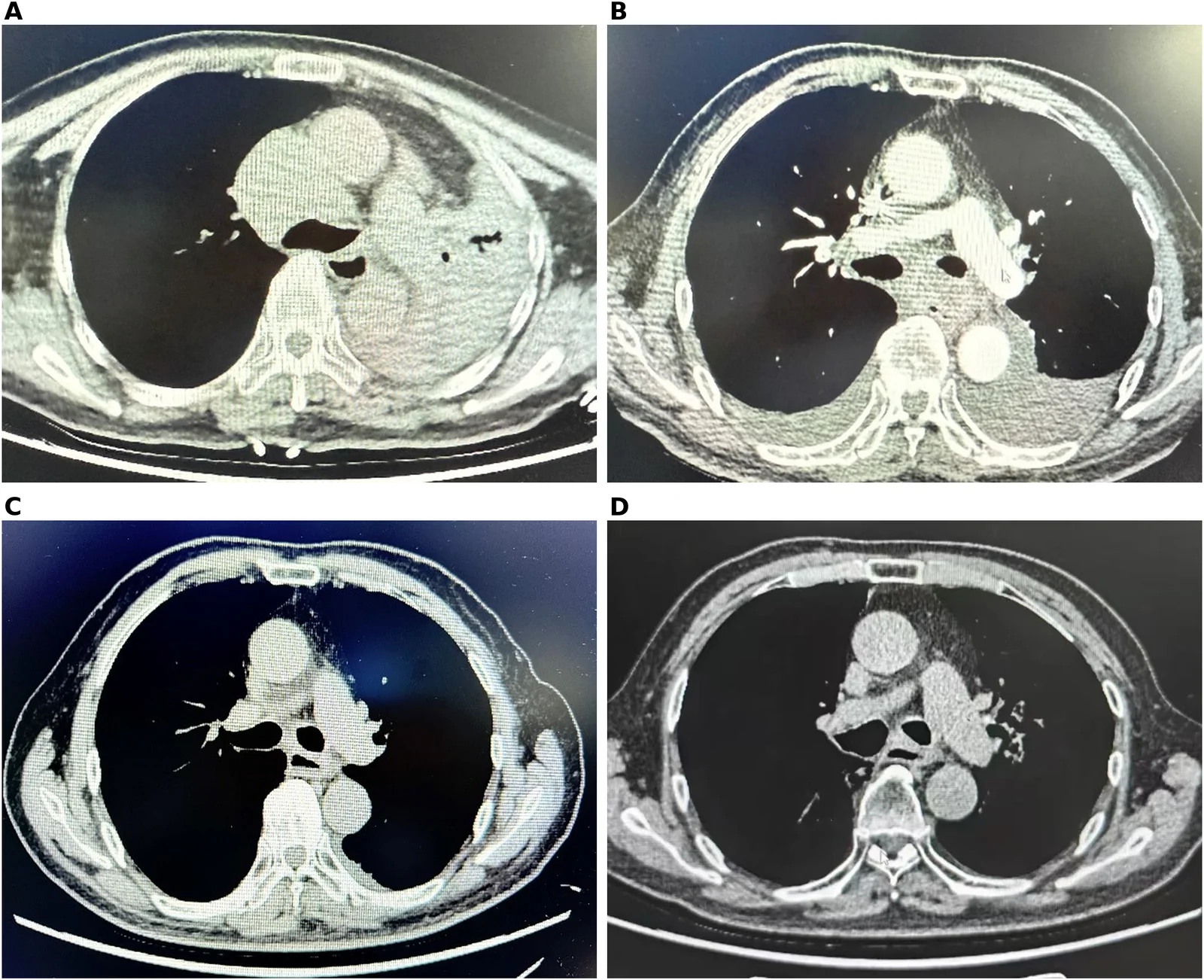
Serial chest CT images demonstrating the radiographic response to treatment. **(A)** Pretreatment chest CT obtained on August 24, 2025, showed a 4.6 × 2.7 cm mediastinal mass causing severe narrowing of the left main bronchus and extensive left lung atelectasis. **(B)** Follow-up chest CT obtained on November 7, 2025, after thoracic radiotherapy and initiation of systemic therapy showed marked reduction of the mediastinal mass to 4.2 × 2.1 cm, improved patency of the left main bronchus, and re-expansion of the left lung. **(C)** Chest CT obtained on April 27, 2026, during ongoing treatment showed further reduction of the mass to 1.9 × 1.2 cm, with sustained airway patency. **(D)** Chest CT obtained on July 20, 2026, showed a residual mediastinal lesion measuring 1.8 × 1.2 cm, with sustained airway patency.

**Table 1:** Clinical timeline of the index case.

Date	Event
August 24, 2025	Presented with cough, wheezing, and progressive dyspnea. Chest CT and PET/CT showed mediastinal lesions with severe airway compression.
August–September 2025	Bronchoscopy, EBUS-TBNA, pathological evaluation, and molecular testing were performed. Multidisciplinary evaluation favored primary HAL, staged as cT2aN3M0, stage IIIB.
September 12–16, 2025	Thoracic IMRT, 15 Gy in 5 fractions (3 Gy/fraction), to the mediastinal nodal lesions and left hilar lesion.
September 18–22, 2025	Second course of thoracic IMRT, 15 Gy in 5 fractions (3 Gy/fraction), to the mediastinal nodal lesions.
September 28, 2025	Tislelizumab 200 mg every 3 weeks was initiated.
October 2025	Lenvatinib 8 mg once daily was added approximately 2 weeks after initiation of tislelizumab.
November 7, 2025	Follow-up CT after thoracic radiotherapy and two cycles of tislelizumab showed a partial response with improved airway patency and normalization of CEA.
April 27, 2026	CT at the cycle 11 assessment showed further tumor reduction.
During treatment	A transient grade 2 elevation in serum transaminases improved after hepatoprotective therapy without treatment interruption or dose reduction.
July 20, 2026	At the latest follow-up, the patient had received 15 cycles of tislelizumab and approximately 9 months of lenvatinib. Treatment with both agents was ongoing, and the patient remained progression-free for nearly 10 months after initiation of tislelizumab.

CEA, carcinoembryonic antigen; EBUS-TBNA, endobronchial ultrasound-guided transbronchial needle aspiration; HAL, hepatoid adenocarcinoma of the lung; IMRT, intensity-modulated radiotherapy.

The patient reported improvement in dyspnea after radiotherapy and the first cycle of systemic therapy. At the last follow-up, he reported that the treatment was tolerable and wished to continue the current regimen.

## Methods

Using the PICOS framework, we included patients with histologically confirmed HAL reported in case reports, case series, or conference abstracts and evaluated their clinicopathologic and molecular characteristics, treatment strategies, responses, and survival. Formal comparators were not required because most reports were uncontrolled. This systematic review was conducted in accordance with the PRISMA 2020 statement. The protocol was registered in PROSPERO (CRD420261440722) on 3 July 2026.

### Eligibility criteria

The inclusion criteria consisted of the following: 1) histopathologically confirmed HAL meeting either the original diagnostic criteria proposed by Ishikura et al. in 1990 (1) or the modified criteria proposed by Haninger et al. in 2014 (5); 2) publication in or after 2000; and 3) case reports, case series, or conference abstracts with sufficient patient-level data to confirm the diagnosis and extract basic demographic information and at least one of the following: stage, treatment, or outcome. The review was limited to publications from 2000 onward to focus on contemporary diagnostic practice and the broader availability of molecular testing.

Exclusion criteria were: 1) review articles, editorials, or other non-original publications; 2) hepatoid adenocarcinoma of non-pulmonary origin, such as gastric or gallbladder origin, or metastatic hepatocellular carcinoma; 3) duplicate or overlapping patient data; and 4) insufficient information for patient-level data extraction.

### Search strategy and study selection

A comprehensive search was conducted in PubMed, Embase, Web of Science, China National Knowledge Infrastructure (CNKI), and Wanfang Data from January 2000 to 4 July 2026. The full search strategies for each database are provided in **Supplementary Table S1**. Reference lists of relevant publications were screened, and forward citation searching was performed when necessary.

Titles, abstracts, and full texts were independently screened by two investigators (JF and YSW). Disagreements were resolved through discussion, with arbitration by a third investigator (XBH) when necessary. The study selection process is summarized in the PRISMA flow diagram (**Figure 3**).

**Figure 3:**
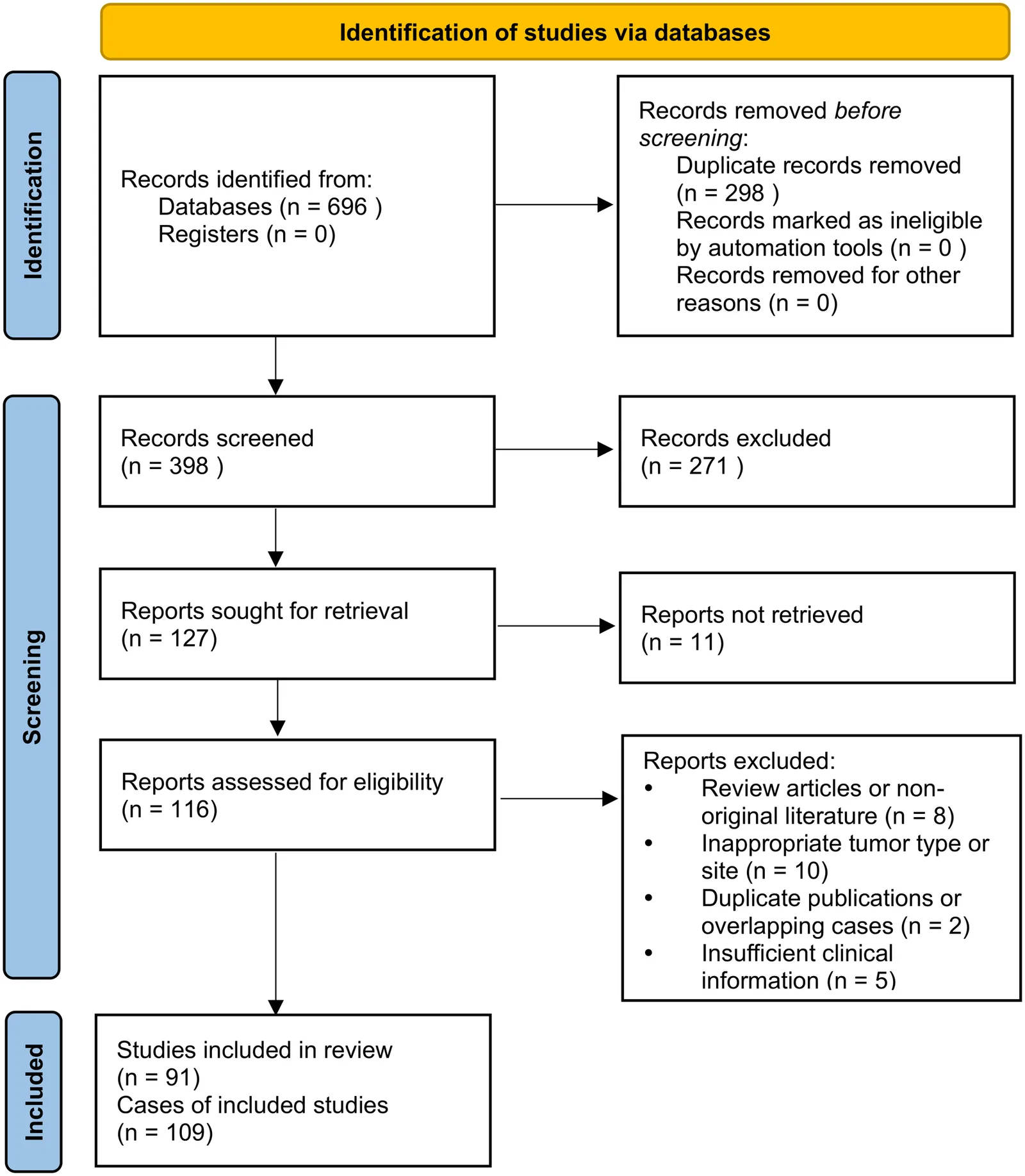
PRISMA 2020 flow diagram of the study selection process.

### Data extraction and quality assessment

Data extraction was independently performed by two investigators (JF and YSW) using a standardized form. Extracted data included patient age and sex, smoking history, tumor location and size, metastatic sites, stage, serum AFP and CEA levels, pathological and IHC findings, molecular testing results, treatment regimens, and prognostic information. Discrepancies were resolved through discussion, with arbitration by a third investigator (XBH) when necessary.

The methodological quality of the included reports was assessed using the Joanna Briggs Institute (JBI) Critical Appraisal Checklists for Case Reports and Case Series, as appropriate. Quality assessment was used to describe the methodological limitations of the included evidence rather than as an exclusion criterion. Detailed results are provided in **Supplementary Table S2**.

### Statistical analysis

All statistical analyses were performed using R version 4.6.1. Survival analyses and visualization were conducted using the survival and survminer packages. Continuous variables were summarized as medians with interquartile ranges (IQRs) and ranges, and categorical variables as numbers and percentages. OS was defined as the time from diagnosis to death from any cause. Patients alive at last follow-up were censored on that date. OS was estimated using the Kaplan–Meier method, and group comparisons were performed using the log-rank test as unadjusted exploratory analyses. Univariable and multivariable Cox proportional hazards regression analyses were performed to evaluate factors associated with OS. Hazard ratios (HRs) were reported with 95% confidence intervals (CIs). All tests were two-sided, and P < 0.05 was considered statistically significant.

## Results

### Clinical characteristics

A total of 91 studies involving 109 cases were included in the systematic review (**Figure 3**) (5–95). Methodological appraisal showed that most case reports adequately described clinical presentation, diagnostic assessment, treatment, and outcomes, although adverse-event reporting was often unclear or absent. In case series, it was often unclear whether all eligible patients were included consecutively and completely (**Supplementary Table S2**). The reported cases were predominantly male (99/109, 91%), with a median age of 63 years (IQR, 53–67; range, 26–83 years; n=109). Smoking history was available for 90 cases, of whom 77 (86%) had a history of smoking. Most cases were reported from China (54/108, 50%), followed by the USA (16/108, 15%), Japan (8/108, 7%), and India (6/108, 6%). Primary tumors were more often right-sided than left-sided (66/105, 63% vs. 39/105, 37%), and the right upper lobe was the most common site among cases with specified lobar location (38/89, 43%). The median maximum tumor diameter was 6.0 cm (IQR, 4.4–8.0; range, 1.0–20.0 cm; n=96).

Most reported cases presented with advanced disease. Stage III–IV disease accounted for 83% of evaluable cases (88/106), including stage IV disease in 47% (50/106). At initial diagnosis, no metastasis was reported in 24% of evaluable cases (24/102), regional nodal involvement in 56% (57/102), and distant metastasis in 49% (50/102). The most common distant metastatic sites were bone (22%, 22/102), brain (12%, 12/102), adrenal gland (10%, 10/102), liver (9%, 9/102), pleura (7%, 7/102), and contralateral lung (7%, 7/102). Serum AFP and CEA were elevated in 60% (49/81) and 59% (24/41) of evaluable cases, respectively. Detailed clinicopathologic characteristics and serum marker findings are summarized in **Supplementary Table S3**. The distributions of several clinical characteristics are presented in **Figure 4**.

**Figure 4:**
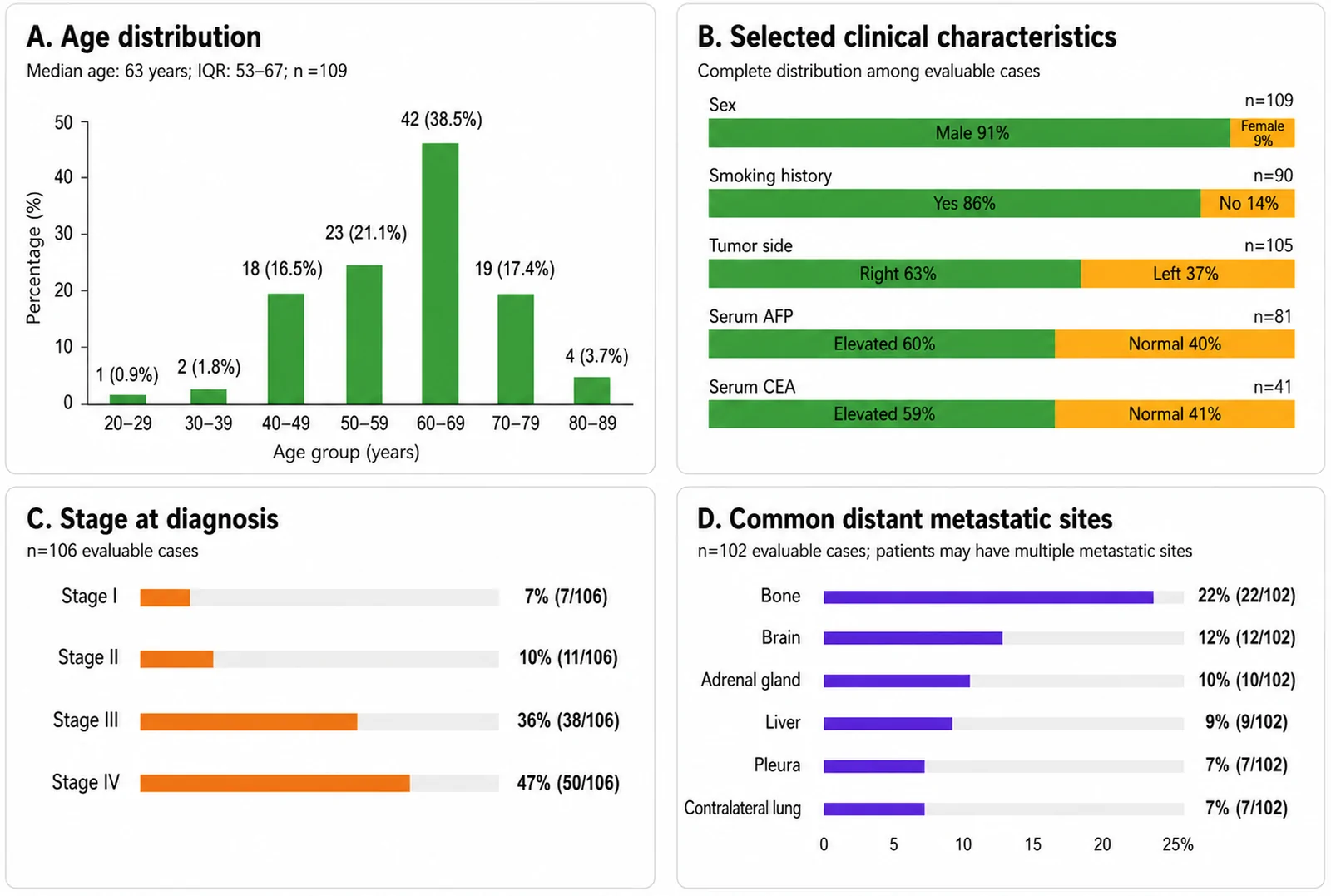
Clinical characteristics of patients with HAL included in the systematic review. **(A)** Age distribution of 109 patients. **(B)** Sex, smoking history, tumor side, and serum AFP and CEA status among evaluable cases. **(C)** Stage at diagnosis among 106 evaluable patients. **(D)** Common distant metastatic sites among 102 evaluable patients. Percentages were calculated using the number of evaluable cases for each variable. Patients could have more than one distant metastatic site.

### IHC and molecular findings

TTF-1 was negative in 64% of evaluable cases (56/87). Among cases with available PD-L1 TPS data, PD-L1 expression was negative (<1%), low (1%–49%), and high (≥50%) in 33% (9/27), 44% (12/27), and 22% (6/27), respectively. Molecular testing results were reported for 49% of cases (53/109), with genomic alterations identified in 36% (19/53). The most frequent alterations were *TP53* mutations (23%, 12/53), *STK11* mutations (9%, 5/53), *KRAS* mutations (8%, 4/53), *SMARCA4* mutations (8%, 4/53), *CDKN2A* mutations (6%, 3/53), *EGFR* mutations (4%, 2/53), and *MET* amplification (4%, 2/53). Detailed pathological features, molecular profiles, treatment categories, and outcomes are summarized in **Supplementary Table S4**.

### Treatment strategies and outcomes

#### Stage I–II HAL

In stage I–II HAL, 16 of 18 cases (89%) underwent surgery. Postoperative recurrence was explicitly reported in 3 cases, occurring at 3, 4, and 9 months after surgery. Reported survival ranged from 5 to >46 months (n=12), and 33% (4/12) survived for at least 24 months.

#### Stage III HAL

Among stage III HAL cases with available treatment data, 57% (21/37) underwent surgery. Postoperative recurrence occurred 3–24 months after surgery. Perioperative treatment included chemotherapy, radiotherapy, immunotherapy, and antiangiogenic therapy. Among 18 patients with available survival data in the surgery group, the Kaplan–Meier-estimated median OS was 23 months. The longest reported survival in this group exceeded 60 months and was observed in a patient who received neoadjuvant camrelizumab plus chemotherapy, followed by surgery and adjuvant camrelizumab plus chemotherapy.

The remaining 43% (16/37) were treated without surgery. In the 13 patients with available survival data, the median OS was 12 months. Reported survival was 1–23 months with chemotherapy alone (n=6), 6–20 months with chemoradiotherapy (n=4), and 7–12 months with radiotherapy alone (n=2). One additional patient received erlotinib followed by chemotherapy, radiotherapy, and antiangiogenic therapy and had an OS of 9 months.

#### Stage IV HAL

In stage IV HAL, the median OS among 39 patients with available survival data was 8 months, and 13% (5/39) had an OS of at least 24 months. First-line systemic treatment regimens and responses are summarized in **Table 2**. Among 17 evaluable patients, chemotherapy alone was the most common first-line treatment (7/17, 41%), with an objective response rate (ORR) of 29% (2/7). The ORR was 60% (3/5) with chemotherapy plus immunotherapy, 50% (1/2) with chemotherapy plus antiangiogenic therapy, and 50% (1/2) with atezolizumab plus bevacizumab. No first-line genotype-directed targeted therapy was reported. One patient with postoperative recurrent KRAS G12C-mutant HAL received sotorasib after chemoradiotherapy and durvalumab, with disease progression after 42 days.

**Table 2:** First-line systemic treatment regimens and outcomes in stage IV HAL cases.

First-line systemic treatment regimen	Specific regimen	Evaluable cases (N)	ORR n/N (%)	SD n/N (%)	PD n/N (%)	PFS/time to progression	OS/follow-up
Chemotherapy	Platinum + gemcitabine; docetaxel + cisplatin/oxaliplatin; S-1; taxane + platinum; carboplatin + pemetrexed	7	2/7 (29%)	2/7 (29%)	3/7 (43%)	4–6 months (n=2)	6–52 months (n=6)
Chemotherapy + immunotherapy	Sintilimab + pemetrexed + carboplatin; carboplatin + paclitaxel + pembrolizumab; camrelizumab + nab-paclitaxel + carboplatin; pemetrexed + carboplatin + pembrolizumab; Durvalumab + tremelimumab + carboplatin + nab-paclitaxel	5	3/5 (60%)	0/5 (0%)	2/5 (40%)	2 cycles to 4 months (n=2)	7–17 months (n=4)
Chemotherapy + antiangiogenic/multikinase therapy	Pemetrexed + cisplatin + bevacizumab; carboplatin + paclitaxel + sorafenib	2	1/2 (50%)	1/2 (50%)	0/2 (0%)	3–5 months (n=2)	11–75 months (n=2)
Immunotherapy + antiangiogenic therapy	Atezolizumab + bevacizumab	2	1/2 (50%)	0/2 (0%)	1/2 (50%)	2–6 cycles (n=2)	4 to >34 months (n=2)
Immunotherapy monotherapy	ICI monotherapy	1	0/1 (0%)	0/1 (0%)	1/1 (100%)	NR	3 months (n=1)
Total	—	17	7/17 (41%)	3/17 (18%)	7/17 (41%)	—	—

This table includes stage IV HAL cases with extractable first-line systemic treatment regimens and response outcomes. Local radiotherapy, surgery, airway intervention, best supportive care, and later-line therapies were not used for treatment classification. Cases receiving local treatment in addition to systemic therapy were classified according to the first-line systemic regimen. PFS/time to progression and OS/follow-up were summarized only for cases with available time data, with the corresponding number shown in each cell.

NR, not reported; ORR, objective response rate; SD, stable disease; PD, progressive disease; ICI, immune checkpoint inhibitor; HAL, hepatoid adenocarcinoma of the lung.

### Survival and prognostic analyses

In 83 patients with available survival data, the median OS was 14 months (95% CI, 9–19 months). For stage I–III disease, surgery was associated with longer median OS (26 vs. 9 months; log-rank P < 0.001). For stage IV disease, chemotherapy was associated with longer median OS (12 vs. 3 months; log-rank P < 0.001) (**Figure 5**). Owing to the observational nature of the data, these survival differences should be interpreted with caution.

**Figure 5:**
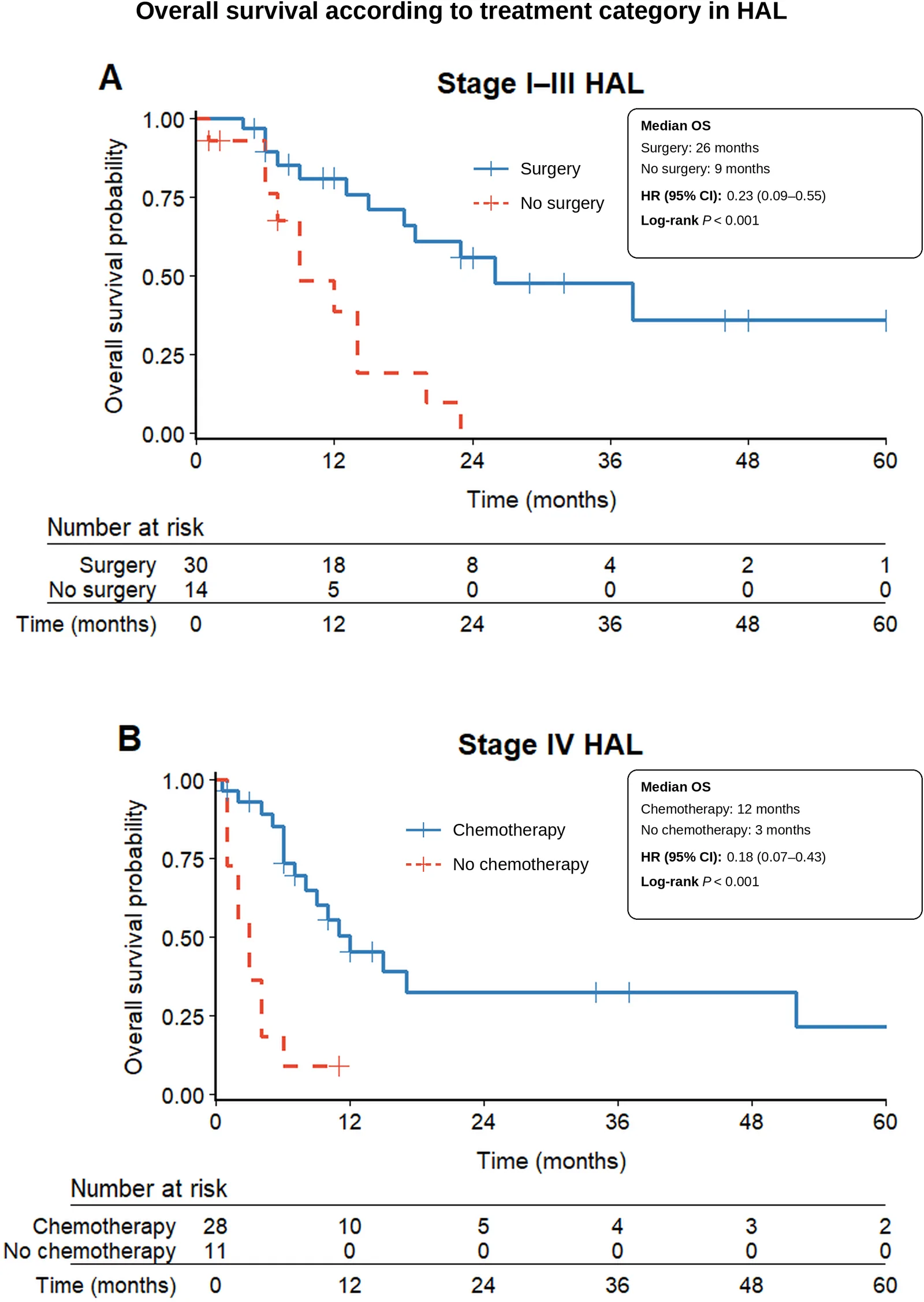
Kaplan–Meier curves of overall survival according to treatment category. **(A)** Comparison between patients with stage I–III HAL whose treatment included surgery (n = 30) and those whose treatment did not (n = 14). **(B)** Comparison between patients with stage IV HAL who received chemotherapy (n = 28) and those who did not (n = 11). P values were calculated using the log-rank test. HRs and 95% CIs were estimated using univariable Cox proportional hazards models.

To identify factors associated with OS, sex, age, smoking history, T, N, and M categories, clinical stage, serum AFP level, and TTF-1 expression were evaluated. In univariable analysis, N2–N3 category (P < 0.001), M1 category (P = 0.047), and stage IV disease (P = 0.026) were associated with shorter OS. Smoking history and TTF-1 expression were not associated with OS (P = 0.405 and P = 0.311, respectively). In multivariable analysis, stage IV disease remained associated with shorter OS (HR, 2.47; 95% CI, 1.18–5.19; P = 0.017) (**Table 3**).

**Table 3:** Univariable and multivariable Cox regression analyses of baseline factors associated with overall survival in patients with HAL.

Characteristics	Total (N)	Univariable analysis		Multivariable analysis	
		P value	Hazard ratio (95% CI)	P value	Hazard ratio (95% CI)
Sex	83	0.109		0.212	
Female			Reference		Reference
Male			2.65 (0.80–8.71)		2.68 (0.57–12.65)
Age, years	83	0.118		0.589	
≤65			Reference		Reference
>65			1.59 (0.89–2.85)		1.23 (0.57–2.66)
Smoking history	69	0.405		—	—
No			Reference		
Yes			1.42 (0.62–3.23)		
T classification	67	0.353		—	—
T1–T2			Reference		
T3–T4			1.42 (0.68–2.97)		
N classification	67	**<0.001**		—	—
N0–N1			Reference		
N2–N3			4.26 (1.96–9.29)		
M classification	76	**0.047**		—	—
M0			Reference		
M1			1.86 (1.01–3.42)		
Stage	83	**0.026**		**0.017**	
I–III			Reference		Reference
IV			1.90 (1.08–3.36)		2.47 (1.18–5.19)
Serum AFP	61	0.174		0.191	
Normal			Reference		Reference
Elevated			0.63 (0.32–1.23)		0.63 (0.31–1.26)
TTF-1 expression	70	0.311		—	—
Negative			Reference		
Positive			1.38 (0.74–2.60)		

Univariable analyses used available cases. The multivariable model included sex, age, stage, and serum AFP and used complete cases.

CI, confidence interval; AFP, alpha-fetoprotein; TTF-1, thyroid transcription factor 1.

Bold values indicate statistical significance (P < 0.05).

## Discussion

Hepatoid adenocarcinoma (HAC) is a rare extrahepatic adenocarcinoma characterized by both glandular and hepatocellular differentiation. HAC has been reported in various organs, including the thymus, pancreas, gallbladder, kidneys, adrenal glands, rectum, ovaries, and uterus (96). The most common site is the stomach (63%), followed by the ovary (10%) and lung (5%) (96).

The reported incidence of HAL is 1.4 per 10 million (4), and most reported patients are older men, often with a history of smoking (97). The male-to-female ratio was 2.5:1 (98). Similar findings were observed in our systematic review: the median age was 63 years, 91% were male, and 86% had a smoking history. HAL has an aggressive clinical course, with 83% presenting with stage III–IV disease at diagnosis (47% stage IV). The median OS was 14 months.

HAL usually presents as a large pulmonary mass, often in an upper lobe (96, 98). In our review, the median maximum tumor diameter was 6.0 cm, and the right upper lobe was the most frequently reported lobe. Diagnosis of HAL is based mainly on histomorphology and IHC. In 2014, Haninger et al. (5) revised the diagnostic criteria for HAL (1): the tumor may consist entirely of hepatoid adenocarcinoma or may contain components of conventional acinar or papillary adenocarcinoma, signet-ring cells, or neuroendocrine carcinoma; and (2) tumoral AFP expression is not required for diagnosis if other markers of hepatic differentiation are expressed. The differential diagnosis of HAL includes metastatic hepatocellular carcinoma, conventional pulmonary adenocarcinoma, germ cell tumors, and hepatoid adenocarcinoma of extrapulmonary origin.

Positive IHC markers reported in HAL include AFP, HepPar-1, CK7, CK8, CK18, CK19, TTF-1, HEA125, MOC31, and EpCAM (2, 5). Reported TTF-1 positivity rates range from 38.5% to 94.1% (2, 99, 100). In our systematic review, TTF-1 was positive in 36% of evaluable cases. This may be related to absent or only focal expression of pulmonary adenocarcinoma-associated markers in HAL with predominant hepatoid differentiation (46, 56). TTF-1 staining is predominantly nuclear in conventional pulmonary adenocarcinoma but often granular and cytoplasmic in HAL (46, 99, 100). In our exploratory survival analysis, TTF-1 expression was not associated with OS (P = 0.311).

Molecular data on HAL remain limited and heterogeneous (98–100). In our review, *TP53*, *STK11*, *KRAS*, *SMARCA4*, and *CDKN2A* were the most frequently reported alterations, whereas potentially actionable EGFR mutations and MET amplification were uncommon. Reported co-mutations involving *TP53*, *STK11*, and *CDKN2A* suggest that altered p53 and cell-cycle regulation, together with PI3K–AKT–mTOR and MAPK signaling, may contribute to HAL pathogenesis (98). Although frequent *KRAS* mutations were reported in one recent cohort, this finding has not been consistently reproduced in other HAL studies (100). Comparative genomic and transcriptomic analyses indicate that HAL is overall closer to lung adenocarcinoma than to hepatocellular carcinoma, while retaining a limited subset of hepatocellular carcinoma-like oncogenic programs (98, 101). These findings warrant further investigation because comprehensive molecular profiling has been performed in only a limited number of cases.

Among the alterations detected in the index case, *TP53* p.R248Q is a missense mutation within the DNA-binding domain of p53. *TP53* mutations can impair normal p53 tumor-suppressive function and have been associated with poorer survival in NSCLC (102). KEAP1 p.I125Mfs*24 is a truncating alteration. Loss of *KEAP1* function can activate NRF2-dependent oxidative-stress responses and promote tumor-cell survival (103). The *B2M* p.E97* truncating alteration may also be relevant to tumor immune biology. B2M is required for stable MHC class I expression and antigen presentation to CD8^+^ T cells. Loss of B2M function can impair tumor-cell recognition and facilitate immune escape (104). CDK12 contributes to genomic stability through transcriptional regulation of DNA damage-response genes (105). The functional significance of the specific *B2M* p.E97*, *CDK12* p.Q695*, and *YAP1* p.R349H variants has not been established.

*NKX2–1* p.V290M was also detected. *NKX2–1* encodes TTF-1 and is a key regulator of pulmonary epithelial lineage identity. Notably, the *NKX2–1* p.V290M alteration coexisted with absent TTF-1 expression in this case. In mouse models of lung adenocarcinoma, deletion of *NKX2–1* drove a lineage switch toward gastric differentiation (106). However, the functional effect of p.V290M has not been established, and current evidence does not show that this variant causes loss of TTF-1 expression or hepatoid differentiation. The tumor also showed a high TMB of 34 mutations/Mb with low PD-L1 expression (TPS 1%–5%). TMB and PD-L1 reflect different aspects of tumor immunogenicity (104), and the clinical significance of this biomarker profile in HAL remains uncertain.

No standard treatment has been established for HAL, and treatment generally follows that for lung adenocarcinoma (4). Surgical resection is generally considered for resectable stage I–III disease. In our review, surgery was associated with longer OS in patients with stage I–III disease, consistent with previous retrospective analyses (2, 3, 98). However, this association may be influenced by selection bias and confounding by indication. Long-term survival has been reported in patients who received neoadjuvant chemoimmunotherapy followed by surgery (16, 22), but these isolated cases are insufficient to define the role of perioperative systemic therapy.

For unresectable stage III HAL, reported treatment approaches include chemotherapy, radiotherapy, and chemoradiotherapy. However, the small number of reported cases precludes meaningful comparison of these approaches. Radiotherapy may also be important for local control and symptom relief. In our patient, thoracic radiotherapy was delivered to relieve airway compromise from locoregional disease, followed by systemic therapy. An OS of 36 months was reported in one patient with *EGFR*-mutant HAL treated with icotinib and osimertinib (28).

For stage IV HAL, platinum-based chemotherapy remains a mainstay of systemic treatment. Chemotherapy was associated with longer OS than no chemotherapy in our analysis, although this association may have been influenced by selection bias and confounding by indication. In a previous comprehensive analysis, taxane–platinum combinations were commonly used, but comparative evidence versus other platinum doublets remains limited (98). In our review, ORRs were numerically higher with first-line combination regimens containing immunotherapy or antiangiogenic therapy than with chemotherapy alone (29, 59, 100). Partial responses to immunotherapy have also been reported despite negative PD-L1 expression (33). Disease control was reported in several cases treated with bevacizumab-containing regimens (11, 13). These findings should be interpreted cautiously owing to the small sample size and clinical heterogeneity.

Multikinase inhibitors used for hepatocellular carcinoma have also been explored in HAL. Sorafenib has shown variable outcomes in individual cases (3, 37). Although lenvatinib achieved disease control for more than 9 months in a patient with recurrent hepatoid adenocarcinoma of the gallbladder, its use in HAL has not previously been reported (107). In our patient, a partial response and sustained disease control were observed after thoracic radiotherapy followed by tislelizumab plus lenvatinib.

HAL is characterized by an aggressive clinical course and poor prognosis, although the biological basis remains unclear. Production of α1-antitrypsin and α1-antichymotrypsin has been proposed as one possible mechanism, as their immunosuppressive and protease-inhibitory properties may promote tumor invasiveness (4). Extensive venous invasion may also contribute to the aggressive behavior of HAL (90). In our multivariable analysis, stage IV disease was independently associated with shorter OS, whereas sex, age, and serum AFP were not. A previous analysis of 65 HAL cases similarly identified clinical stage as the only factor associated with OS (108). Other studies have reported an association between surgery and longer survival (3, 98). Treatment variables were not included in our prognostic model because treatment selection varied substantially by disease stage and was susceptible to confounding by indication.

The prognostic value of serum AFP remains uncertain. Some studies reported an association between elevated pretreatment AFP or persistent AFP elevation at recurrence and shorter survival, whereas others found no significant association between AFP and OS (3, 108). Serum AFP was likewise not associated with OS in our analysis.

This study has several limitations. In the index case, thoracic lesions regressed after radiotherapy followed by systemic therapy, and no measurable HAL lesion outside the radiation field was available for independent assessment of the response to systemic therapy. Most included studies were case reports or small case series, and the available clinical, molecular, treatment, and follow-up data were often incomplete. Comparability across reported cases was limited by differences in diagnostic criteria, staging systems, treatment strategies, and reporting quality. In addition, the small sample size and observational nature of the available evidence increased the risk of selection and publication bias and limited adjustment for potential confounders. Therefore, the survival and treatment findings should be interpreted with caution.

## Conclusion

HAL is a rare and aggressive pulmonary malignancy most often reported in older men. Diagnosis relies on integration of radiologic, morphologic, and IHC findings, with careful exclusion of an extrapulmonary primary. HAL generally has a poor prognosis. Clinical stage was associated with OS in our analysis, whereas the prognostic value of serum AFP remains uncertain. No standard of care exists for HAL, and treatment is generally extrapolated from lung adenocarcinoma practice. The roles of genotype-directed targeted therapy and immunotherapy warrant further investigation.

## Data Availability

The original contributions presented in the study are included in the article/**Supplementary Material**. Further inquiries can be directed to the corresponding author.

## References

[1] Ishikura H Kanda M Ito M Nosaka K Mizuno K . Hepatoid adenocarcinoma: a distinctive histological subtype of alpha-fetoprotein-producing lung carcinoma. Virchows Arch A Pathol Anat Histopathol. (1990) 417:73–80. doi: 10.1007/BF01600112 1694332

[2] Mao J-X Liu C Zhao Y-Y Ding G-S Ma J-Q Teng F . Merged hepatopulmonary features in hepatoid adenocarcinoma of the lung: a systematic review. Am J Transl Res. (2021) 13:898–922. PMC8014347 33841629

[3] Hou Z Xie J Zhang L Dai G Chen Y He L . Hepatoid adenocarcinoma of the lung: a systematic review of the literature from 1981 to 2020. Front Oncol. (2021) 11:702216. doi: 10.3389/fonc.2021.702216 34422656 PMC8377348

[4] Chen X Wei H Sun Q Zhao H Yan P Gong C . Progress in diagnosis and treatment of hepatoid adenocarcinoma of the lung. Chin J Clin (Electron Edition). (2024) 18:83–6. doi: 10.3877/cma.j.issn.1674-0785.2024.01.013. [Article in Chinese].

[5] Haninger DM Kloecker GH Bousamra IM Nowacki MR Slone SP . Hepatoid adenocarcinoma of the lung: report of five cases and review of the literature. Mod Pathol. (2014) 27:535–42. doi: 10.1038/modpathol.2013.170 24030743

[6] Talat F Talpur AS Ahmad B Mahjoub M Raheela F Ramesh AS . Hepatoid adenocarcinoma of the lung: a case report on a rare medical condition. Cureus. (2026) 18:e102211. doi: 10.7759/cureus.102211 41737117 PMC12927955

[7] Kapes G Merizaj XB Gakidis I Chantziantoniou C Michos P . A mandibular metastasis as the initial presentation of hepatoid adenocarcinoma of the lung: a case report. Cureus. (2026) 18:e101740. doi: 10.7759/cureus.101740 41704974 PMC12908687

[8] Fernández Aceñero MJ Acedo V Barreiro P Negueruela M Nielsen L . Primary hepatoid adenocarcinoma of the lung: clues for cytological diagnosis. Cytopathology. (2025) 36:512–5. doi: 10.1111/cyt.13494 40229866

[9] Zahfir I Oueriagli SN Aboussabr M Sahel OA Benameur Y Doudouh A . 18F-FDG PET/CT in lung hepatoid adenocarcinoma. Indian J Nucl Med. (2025) 40:314–5. doi: 10.4103/ijnm.ijnm_71_25 41281175 PMC12633803

[10] Wang S Li M Chen H Li J Zeng Q . FDG PET/CT in hepatoid adenocarcinoma of the lung. Clin Nucl Med. (2016) 41:e340–e341. doi: 10.1097/RLU.0000000000001231 27124679

[11] Wang X Wei N Yang H Wang X Zhang X Zhang Q . Long term survival of advanced hepatoid adenocarcinoma of lung secondary to idiopathic pulmonary fibrosis: a case report. Front Oncol. (2025) 15:1487334. doi: 10.3389/fonc.2025.1487334 39968074 PMC11832387

[12] Khabbaza R Shawish A Mahmoud A Houreih MA Al-Janabi MH Hassan F . A rare case of hepatoid adenocarcinoma of the lung initially presenting as a painful skin nodule. J Surg Case Rep. (2025) 2025:rjaf708. doi: 10.1093/jscr/rjaf708 40927507 PMC12416816

[13] Hua YY Huang SY . Case report of primary hepatoid lung carcinoma in a 45-year-old woman. J Cancer Res Pract. (2025) 12:98–103. doi: 10.4103/ejcrp.eJCRP-D-25-00013

[14] Harrell D Twito JR Perkins M Krishnan P Holladay RC Chambers DM . Hepatoid adenocarcinoma of the lung. Chest. (2025) 168:A6026–7. doi: 10.1016/j.chest.2025.07.337742574925

[15] Mo Y-J Lin L-N Tao J-L Zhang T Zhang J-H . Hepatoid adenocarcinoma of the lung: a case report. World J Clin cases. (2024) 12:4813–9. doi: 10.12998/wjcc.v12.i21.4813 39070823 PMC11235510

[16] Huang X Zhu L Pan W Hu J . Long survival after neoadjuvant and adjuvant camrelizumab plus chemotherapy and surgery in a patient with hepatoid adenocarcinoma of the lung: A case report. Hum Vaccin Immunother. (2024) 20:2342133. doi: 10.1080/21645515.2024.2342133 38659098 PMC11057579

[17] Uruga H Miyamoto A Fujii T Okuma T Mitsumura T Tamaoka M . A case of hepatoid adenocarcinoma of the lung harboring KRAS G12C responded favorably to sotorasib. Pathol Int. (2024) 74:546–51. doi: 10.1111/pin.13464 39016668

[18] Cai C Zhang N Wang M Wang L Zhao H Zhang X . Primary hepatoid adenocarcinoma of the lung with extremely elevated serum AFP: a case report and literature review. Front Oncol. (2024) 14:1448219. doi: 10.3389/fonc.2024.1448219 39421450 PMC11484443

[19] Wang B Pan L-Y Shen X-Y Chen P-C . Hepatoid adenocarcinoma of the lung: A case report. Asian J Surg. (2024) 47:4914–5. doi: 10.1016/j.asjsur.2024.05.193 38824023

[20] Xu K Gao J Feng L Fang Y Tang X . A case report of pulmonary hepatoid adenocarcinoma: promoting standardized diagnosis and treatment of the rare disease. Front Immunol. (2023) 14:1203876. doi: 10.3389/fimmu.2023.1203876 37292208 PMC10244673

[21] Sun X Liu J Hu T Wu Y Zhang H . A rare case of hepatoid adenocarcinoma of the lung. Clin Respir J. (2023) 17:1368–71. doi: 10.1111/crj.13724 38071759 PMC10730454

[22] Chen K Yu Y Huang Z Gong L Fan Y . Complete response of a locally advanced pulmonary hepatoid adenocarcinoma patient to perioperative XELOX-containing chemoimmunotherapy. Genes Dis. (2024) 11:1–3. doi: 10.1016/j.gendis.2022.12.006 37588195 PMC10425740

[23] Huang L Chen C Sun Q Yu Z Wang X Wang X . Primary hepatoid adenocarcinoma of the lung in patient with silicosis: a case report and literature review. Front Oncol. (2024) 14:1380717. doi: 10.3389/fonc.2024.1380717 39534092 PMC11554529

[24] Li Y Liu L Wang D Tan X Sui Y Yang H . Hepatoid adenocarcinoma of the lung with PD-L1 expression and the response to anti-PD-1 therapy: a case report. Front Oncol. (2023) 13:1257931. doi: 10.3389/fonc.2023.1257931 38074660 PMC10702343

[25] Bonis A Dell’Amore A Verzeletti V Melan L Zambello G Nardocci C . Hepatoid adenocarcinoma of the lung: A review of the most updated literature and a presentation of three cases. J Clin Med. (2023) 12:1411. doi: 10.3390/jcm12041411 36835946 PMC9964907

[26] Chen Z Ding C Zhang T He Y Jiang G . Primary hepatoid adenocarcinoma of the lung: a systematic literature review. Onco Targets Ther. (2022) 15:609–27. doi: 10.2147/OTT.S364465 35676912 PMC9167841

[27] Sun H Li X Zhang J Liu Y . Clinicopathological features and genomic profiles of hepatoid adenocarcinoma of the lung: Report of four cases. Pathol Res Pract. (2022) 229:153652. doi: 10.1016/j.prp.2021.153652 34826742

[28] Chen H Wang W Li X Xu C Du K Zhu Y . Hepatoid adenocarcinoma of the lung with EGFR mutation and the response to tyrosine kinase inhibitors. J Thorac Oncol. (2019) 14:e217–e219. doi: 10.1016/j.jtho.2019.04.032 31558230

[29] Lagos GG Feldman JL Saqi A Shu CA . Hepatoid adenocarcinoma of the lung responsive to frontline combination chemotherapy with immunotherapy: case report. JTO Clin Res Rep. (2021) 2:100130. doi: 10.1016/j.jtocrr.2020.100130 34589989 PMC8474391

[30] Wang W Li G . Radiotherapy of pulmonary hepatoid adenocarcinoma with intrahepatic hemangioma: a case report. Onco Targets Ther. (2020) 13:11947–55. doi: 10.2147/OTT.S275340 33239894 PMC7682605

[31] Yang K Jiang H Li Q . Primary pulmonary hepatoid adenocarcinoma: A case report and review of the literature. Med (Baltimore). (2019) 98:e15053. doi: 10.1097/MD.0000000000015053 30946350 PMC6455993

[32] Wang C Xu G Wu G Chen Z Sun Z Zheng P . Hepatoid adenocarcinoma of the lung metastasizing to the gingiva. Onco Targets Ther. (2019) 12:8765–8. doi: 10.2147/OTT.S222974 31749622 PMC6817769

[33] Basse V Schick U Guéguen P Le Maréchal C Quintin-Roué I Descourt R . A mismatch repair–deficient hepatoid adenocarcinoma of the lung responding to anti–PD-L1 durvalumab therapy despite no PD-L1 expression. J Thorac Oncol. (2018) 13:e120–e122. doi: 10.1016/j.jtho.2018.03.004 29935850

[34] Carlinfante G Foschini MP Pasquinelli G Scotti R Cavazza A . Hepatoid carcinoma of the lung: a case report with immunohistochemical, ultrastructural and in-situ hybridization findings. Histopathology. (2000) 37:88–9. doi: 10.1046/j.1365-2559.2000.00955-5.x 10931226

[35] Kitada M Ozawa K Sato K Matsuda Y Hayashi S Tokusashi Y . Alpha-fetoprotein-producing primary lung carcinoma: A case report. World J Surg Oncol. (2011) 9:47. doi: 10.1186/1477-7819-9-47 21554678 PMC3103442

[36] Shaib W Sharma R Mosunjac M Farris AB El Rayes B . Hepatoid adenocarcinoma of the lung: a case report and review of the literature. J Gastrointest Cancer. (2014) 45:99–102. doi: 10.1007/s12029-013-9558-7 24408270

[37] Gavrancic T Park YHA . A novel approach using sorafenib in alpha-fetoprotein–producing hepatoid adenocarcinoma of the lung. J Natl Compr Canc Netw. (2015) 13:387–91. doi: 10.6004/jnccn.2015.0054 25870375

[38] Ayub A Nunez Lopez O Booth A Okereke I . Pulmonary hepatoid adenocarcinoma. J Thorac Cardiovasc Surg. (2019) 158:e139–e140. doi: 10.1016/j.jtcvs.2019.06.023 31358339

[39] Li J Qi H Xu B Zhao J Gao H Ma X . Genomic profiles of a patient of pulmonary hepatoid adenocarcinoma with high AFP level: a case report. Front Oncol. (2019) 9:1360. doi: 10.3389/fonc.2019.01360 31921629 PMC6917606

[40] Laraqui Houssein Y Derqaoui S Khouloud R Jahid A Zouaidia F Bernoussi Z . Hepatoid adenocarcinoma of the lung: A rare form of lung cancer. Clin Pathol. (2022) 15:2632010X221088961. doi: 10.1177/2632010X221088961 35434621 PMC9005818

[41] Iino K Ohta Y Tamura M Sato H Katayanagi K Kurumaya H . A resected case of alpha-fetoprotein-producing hepatoid adenocarcinoma of the lung. Haigan. (2003) 43:29–34. doi: 10.2482/haigan.43.29. [Article in Japanese].

[42] Alonso-Gonzalo L Oblitas CM Cebollero-Presmanes M Vieru CM García-Leoni ME . Hepatoid adenocarcinoma of the lung: a rare case report. Galicia Clin. (2025) 86:27–9. doi: 10.22546/aeebcq8442689263

[43] Ivan M Koss M Chang C-F . HEPATOID ADENOCARCINOMA OF THE LUNG. Chest. (2007) 132:690A. doi: 10.1378/chest.132.4_MeetingAbstracts.69019292919

[44] Feng GW Hu JJ Li B . Detection of pulmonary hepatoid adenocarcinoma with elevated alpha-fetoprotein by 18F-FDG PET/CT: a case report. J Diagn Concepts Pract. (2013) 12:231–3. doi: 10.16150/j.1671-2870.a0511. [Article in Chinese].

[45] Liu HY Wang XM Cheng ZQ Peng Q Sun X Hu J . Clinicopathologic observation of primary hepatoid adenocarcinoma of the lung. Chin J Clin (Electron Edition). (2014) 8:2608–12. doi: 10.3877/cma.j.issn.1674-0785.2014.14.010 [Article in Chinese].

[46] Grossman K Beasley MB Braman S . Carcinoma with hepatoid features: A rare form of lung cancer. Chest. (2014) 145:318A. doi: 10.1378/chest.180001619292919

[47] Al-Najjar H Evison M Doran H Booton R Taylor P . Primary pulmonary hepatoid adenocarcinoma: A case report. Cancer Treat Commun. (2015) 4:172–3. doi: 10.1016/j.ctrc.2015.10.00142574925

[48] Matsuda Y Kitada M Hayashi S Ishibashi K Yasuda S Ohsaki Y . A case report of hepatoid adenocarcinoma of the lung. Haigan. (2015) 55:161–5. doi: 10.2482/haigan.55.161. [Article in Japanese].

[49] Esa NYM Zamrud R Bakar NS Eezamuddeen MN Hamdan MF . Is it liver or lung cancer? An intriguing case of lung adenocarcinoma with hepatoid differentiation. Proc Singap Healthc. (2018) 27:55–8. doi: 10.1177/2010105817716185

[50] Chen L Han X Gao Y Zhao Q Wang Y Jiang Y . Anti-PD-1 therapy achieved disease control after multiline chemotherapy in unresectable KRAS-positive hepatoid lung adenocarcinoma: a case report and literature review. Onco Targets Ther. (2020) 13:4359–64. doi: 10.2147/OTT.S248226 32547068 PMC7245476

[51] Chen J-X Lyu L-L Zhu W-H Chen X-Y Zheng L . Hepatoid adenocarcinoma of the lung accompanied with multiple systemic metastases. Chin Med J (Engl). (2021) 134:237–8. doi: 10.1097/CM9.0000000000000963 32826610 PMC7817322

[52] Miyama Y Fujii T Murase K Takaya H Kondo F . Hepatoid adenocarcinoma of the lung mimicking metastatic hepatocellular carcinoma. Autopsy Case Rep. (2020) 10:e2020162. doi: 10.4322/acr.2020.162 33344280 PMC7703083

[53] Hou Q Zhang YP Zhang YX Li W . A case of primary hepatoid adenocarcinoma of the lung. J Diagn Pathol. (2017) 24:473–5. doi: 10.3969/j.issn.1007-8096.2017.06.020 [Article in Chinese].

[54] Zhong MY Liu B . Hepatoid adenocarcinoma of the lung: a case report and literature review. J Capital Med Univ. (2015) 36:515–6. doi: 10.3969/j.issn.1006-7795.2015.03.037. [Article in Chinese].

[55] He XY Gao ZY Xu H Hu TT Liang W Sun YC . A case of hepatoid adenocarcinoma of the lung. Chin J Clin Oncol. (2020) 47:1025–6. doi: 10.3969/j.issn.1000-8179.2020.19.637. [Article in Chinese].

[56] Pasricha S Grover S Kamboj M Bansal D Batra U Gupta G . Hepatoid adenocarcinoma of lung: a diagnostic challenge—series of six cases with histopathological, predictive molecular and PD.L1 assessment. Indian J Pathol Microbiol. (2021) 64:128–31. doi: 10.4103/IJPM.IJPM_334_20 33433422

[57] Meyers M . S3044 Hepatoid adenocarcinoma—a mimicker of hepatocellular carcinoma. Am J Gastroenterol. (2022) 117:e1965–6. doi: 10.14309/01.ajg.0000868816.32093.4239449940

[58] Yao Y Guan X Bao G Liang J Li T Zhong X . Whole-exome sequencing and bioinformatics analysis of a case of non-alpha-fetoprotein-elevated lung hepatoid adenocarcinoma. Front Pharmacol. (2022) 13:945038. doi: 10.3389/fphar.2022.945038 36091765 PMC9462446

[59] Arai H Sasada S Kaburaki K Ochiai R Masuda W . A case of advanced non-small cell lung cancer with hepatocellular carcinoma-like features responding to a combination of durvalumab, tremelimumab, carboplatin, and nab-paclitaxel. Cureus. (2025) 17:e79740. doi: 10.7759/cureus.79740 40161114 PMC11954152

[60] Cao YA Long NZ Peng CS Lu P Xia J Wang W . A case of lung adenocarcinoma with liver metastasis and elevated alpha-fetoprotein. Chin J Clin Oncol. (2008) 35:420. doi: 10.3969/j.issn.1000-8179.2008.07.019 [Article in Chinese].

[61] Ding QJ Deng Q Li KM Pan YN . A case of hepatoid adenocarcinoma of the lung. Chin J Thorac Cardiovasc Surg. (2019) 35:700–1. doi: 10.3760/cma.j.issn.1001-4497.2019.11.014. [Article in Chinese].

[62] Xu YJ Lin N Fan DG Huang ZX . Two cases of AFP-negative hepatoid adenocarcinoma of the lung. Chin J Clin Exp Pathol. (2020) 36:1495–6. doi: 10.13315/j.cnki.cjcep.2020.12.033 [Article in Chinese].

[63] Fan XL Zhang D . A case of hepatoid adenocarcinoma of the lung. Chin J Thorac Cardiovasc Surg. (2019) 35:373–4. doi: 10.3760/cma.j.issn.1001-4497.2019.06.01534816785. [Article in Chinese].

[64] Li Y Miao JJ Wu JC Ji SJ . Alpha-fetoprotein-negative hepatoid adenocarcinoma of the lung: a case report and literature review. J Int Oncol. (2019) 46:574–6. doi: 10.3760/cma.j.issn.1673-422X.2019.09.01430704229. [Article in Chinese].

[65] Yu DZ Wang LH Ma JJ Wang ZY Gao Q Yang ZK . Hepatoid adenocarcinoma of the lung invading the spleen: a case report. J Clin Surg. (2020) 28:739–40. [Article in Chinese]. doi: 10.3969/j.issn.1005-6483.2020.08.013

[66] Shen C Duan XY . 18F-FDG PET/CT imaging of hepatoid adenocarcinoma of the lung: a case report. Int J Radiat Med Nucl Med. (2021) 45:604–6. doi: 10.3760/cma.j.cn121381-202008028-0009530704229. [Article in Chinese].

[67] Zeng ZY Qu XL Zhang FY Li QW Xiao WH . A case of central hepatoid adenocarcinoma of the lung. Chin Clin Oncol. (2023) 28:478–80. [Article in Chinese]. doi: 10.3969/j.issn.1009-0460.2023.05.014

[68] Cao DM Lin MF Chen WX Zhou S Chen CL Chen GB . 18F-FDG PET/CT findings of hepatoid adenocarcinoma of the lung: report of 3 cases. Chin J Med Imaging Technol. (2023) 39:310–2. [Article in Chinese]. doi: 10.13929/j.issn.1003-3289.2023.02.038

[69] Liu GR Li GH Li WB Zeng RB . Hepatoid adenocarcinoma of the lung: a case report and literature review. J Clin Pulm Med. (2023) 28:1776–7. doi: 10.3969/j.issn.1009-6663.2023.11.03426136947. [Article in Chinese].

[70] Zhang K Liu Q Liu B Lin D . Primary alpha-fetoprotein-positive hepatoid adenocarcinoma of the lung: a case report. Chin J Tuberculosis Respir Dis. (2023) 46:700–7. doi: 10.3760/cma.j.cn112147-20221103-00874 37402661. [Article in Chinese].

[71] Zhang YY Feng LJ Yang Q . Hepatoid adenocarcinoma of the lung: a case report. J Pract Radiol. (2023) 39:1049–50. doi: 10.3969/j.issn.1002-1671.2023.06.04939070823. [Article in Chinese].

[72] Li Q Liu ZF Zhang EN Fu AQ . Hepatoid adenocarcinoma of the lung with MET amplification: a case report. Chin J Metastatic Cancer. (2023) 6:151–4. doi: 10.3760/cma.j.cn101548-20210301-0003234816785. [Article in Chinese].

[73] Zou LQ Chen QX Wang Y Cheng X Liu Y Li FN . Primary hepatoid adenocarcinoma of the lung: a clinicopathological analysis and literature review. J Diagn Pathol. (2023) 30:59–62. doi: 10.3969/j.issn.1007-8096.2023.01.014. [Article in Chinese].

[74] Hong JD Luo TB Shi L Pan Y Li F Zhang Y . Multimodality therapy of a patient with SMARCA4-deficient hepatoid adenocarcinoma of the lung. J Chin Oncol. (2025) 31:175–9. doi: 10.11735/j.issn.1671-170X.2025.02.B014 [Article in Chinese].

[75] Bai CG Liu XH Yu YW Zhang SM Ma DL . Hepatoid adenocarcinoma of the lung: a case report and literature review. Chin J Clin Exp Pathol. (2006) 22:246–8. doi: 10.3969/j.issn.1001-7399.2006.02.037 [Article in Chinese].

[76] Seddon JE Jayarao M Donahue JE Toms SA . Brain metastasis secondary to hepatoid adenocarcinoma of the lung. Interdiscip Neurosurg. (2021) 24:101085. doi: 10.1016/j.inat.2020.10108542574925

[77] Shahrudin MS Wong YS Hasbullah HH Alias AB Musa AN . Hepatoid adenocarcinoma of the lung with hard palate metastasis: review of a rare presentation of lung cancer. Respirology. (2023) 28:326.

[78] Cavalcante LB Felipe-Silva A de Campos FPF Martines JADS . Hepatoid adenocarcinoma of the lung. Autops Case Rep. (2013) 3:5–14. doi: 10.4322/acr.2013.002 31528592 PMC6671883

[79] Shao Y Zhong DS Wang D Ma L . Hepatoid adenocarcinoma of the lung: a case report. Int J Clin Exp Pathol. (2016) 9:4067–72. 39421320

[80] Lucas C Costa J Ribeiro P Rodrigues A . Hepatoid adenocarcinoma of the lung. Galicia Clín. (2018) 79:45–6. doi: 10.22546/48/152342655252

[81] Ruiz Cerrato D Wang Memoli JS Lee W . Hepatoid adenocarcinoma of the lung with normal alpha-fetoprotein level. Am J Respir Crit Care Med. (2018) 197:A4018. doi: 10.1164/ajrccm-conference.2018.197.1_MeetingAbstracts.A4018

[82] Terracciano LM Glatz K Mhawech P Vasei M Lehmann FS Vecchione R . Hepatoid adenocarcinoma with liver metastasis mimicking hepatocellular carcinoma: an immunohistochemical and molecular study of eight cases. Am J Surg Pathol. (2003) 27:1302–12. doi: 10.1097/00000478-200310000-00002 14508391

[83] Genova S Dikov D Peshev Z Khekimov K Vuzhev Z Khristova S . Hepatoid adenocarcinoma of the lung: a case report. Khirurgiia (Sofiia). (2003) 59:45–7. [Article in Bulgarian]. 15641539

[84] Kuan K Khader SN El Hussein S . Hepatoid adenocarcinoma of the lung. Diagn Cytopathol. (2019) 47:831–3. doi: 10.1002/dc.24195 31041840

[85] Hayashi Y Takanashi Y Ohsawa H Ishii H Nakatani Y . Hepatoid adenocarcinoma in the lung. Lung Cancer. (2002) 38:211–4. doi: 10.1016/s0169-5002(02)00214-3 12399135

[86] No EJ Hur JW Lee SS Lee YM Lee HP Kim JI . A case of alpha-fetoprotein-producing adenocarcinoma of the lung. Tuberc Respir Dis. (2006) 61:70–3. doi: 10.4046/trd.2006.61.1.7012631605. [Article in Korean].

[87] Wang PC Chang HY Hsiue TR . Alpha-fetoprotein-producing hepatoid adenocarcinoma originating in the lung: a case report. Thorac Med. (2008) 23:205–10. doi: 10.29806/tm.200806.000938824023. [Article in Chinese].

[88] Papatsimpas G Kamposioras K Goula K Papaparaskeva K Loukides S Kotoulas C . Hepatoid pancoast tumor. A case report and review of the literature. Lung Cancer. (2012) 77:239–45. doi: 10.1016/j.lungcan.2012.05.102 22677427

[89] Mokrim M Belbaraka R Allaoui M Kairaouani M Mahassini N Tahri A . Hepatoid adenocarcinoma of the lung: a case report and literature review. J Gastrointest Cancer. (2012) 43:S125–7. doi: 10.1007/s12029-011-9318-5 21894458

[90] Che YQ Wang S Luo Y Wang JB Wang LH . Hepatoid adenocarcinoma of the lung: presenting mediastinal metastasis without transfer to the liver. Oncol Lett. (2014) 8:105–10. doi: 10.3892/ol.2014.2064 24959228 PMC4063569

[91] Sun JN Zhang BL Li LK Yu HY Wang B . Hepatoid adenocarcinoma of the lung without production of alpha-fetoprotein: a case report and review of the literature. Oncol Lett. (2016) 12:189–94. doi: 10.3892/ol.2016.4559 27347123 PMC4906620

[92] Nakashima K Okagawa T . Hepatoid adenocarcinoma of the lung with high serum AFP and CEA values: report of a case. Kyobu Geka. (2018) 71:76–9. [Article in Japanese]. 29483483

[93] Shi Y-F Lu J-G Yang Q-M Duan J Lei Y-M Zhao W . Primary hepatoid adenocarcinoma of the lung in yungui plateau, China: a case report. World J Clin cases. (2019) 7:1711–6. doi: 10.12998/wjcc.v7.i13.1711 31367631 PMC6658376

[94] El Khoury A El Khoury M De Luca R . Immunotherapeutic approach to a case of advanced hepatoid adenocarcinoma of the lung. memo. (2019) 12:272–7. doi: 10.1007/s12254-019-0499-030311153

[95] Cancela Díez B Gómez de Rueda F Valdivia Bautista J Aneiros Fernández JF Jiménez Morales A . Review and case report: hepatoid adenocarcinoma of the lung treated with nivolumab metastatizing to the skin. Eur J Clin Pharm: Atención Farmacéutica. (2019) 21:149–54.

[96] Metzgeroth G Ströbel P Baumbusch T Reiter A Hastka J . Hepatoid adenocarcinoma - review of the literature illustrated by a rare case originating in the peritoneal cavity. Onkologie. (2010) 33:263–9. doi: 10.1159/000305717 20502062

[97] Wang W Li G . Incidence and prognostic factors of hepatoid adenocarcinoma: a population-based analysis. Transl Cancer Res. (2020) 9:5401–10. doi: 10.21037/tcr-20-1126 35117905 PMC8798184

[98] Deng H Wang L Li Z Zhan T Huang L . Optimal treatment strategies for hepatoid adenocarcinoma of the lung: insights from a comprehensive analysis. BMC Cancer. (2024) 24:948. doi: 10.1186/s12885-024-12682-z 39095810 PMC11297620

[99] Nezami BG Fridland S Dos Santos LS Jennings L Sukhanova M Lu X . Hepatoid adenocarcinoma of the lung: clinicopathologic and molecular analysis of 17 cases. Virchows Arch. (2026) 489:91–101. doi: 10.1007/s00428-026-04558-3 42096087 PMC13368951

[100] Fave T Nguyen J Descourt R Quere G Dhamelincourt E Simaki A . Real-life experience of rare hepatoid adenocarcinomas of the lung: a large retrospective French cohort. Clin Lung Cancer. (2025) 26:e432–40. doi: 10.1016/j.cllc.2025.05.003 40537320

[101] Fridland S Amgad M Chen J Sukhanova M Santos LS dos Jennings L . 1621 hepatoid adenocarcinoma of lung retains selective hepatic oncogenic programs within a lung-like transcriptome. Lab Invest. (2026) 106:105923. doi: 10.1016/j.labinv.2025.10592342574925

[102] Zhang C Yang C Shi Q . Effects of Tp53 gene mutations on the survival of non-small cell lung cancer (NSCLC); a short review. Cancer Manag Res. (2025) 17:65–82. doi: 10.2147/CMAR.S495006 39830995 PMC11742633

[103] Paredes R Borea R Drago F Russo A Nigita G Rolfo C . Genetic drivers of tumor microenvironment and immunotherapy resistance in non-small cell lung cancer: the role of KEAP1, SMARCA4, and PTEN mutations. J Immunother Cancer. (2025) 13:e012288. doi: 10.1136/jitc-2025-012288 40764107 PMC12336572

[104] Han J Yang Z Zhao H . Lung cancer immunotherapy in 2025: where we stand and what comes next? Front Immunol. (2026) 16:1728163. doi: 10.3389/fimmu.2025.1728163 41624891 PMC12855455

[105] Dudkiewicz-Garbicz J Włodarski PK . CDK12 and CDK13 in oncology: from RNA regulation to therapeutic targeting. Cell Oncol. (2026) 49:13. doi: 10.1007/s13402-025-01131-z 41491919 PMC12769584

[106] Zewdu R Mehrabad EM Ingram K Fang P Gillis KL Camolotto SA . An NKX2-1/ERK/WNT feedback loop modulates gastric identity and response to targeted therapy in lung adenocarcinoma. eLife. (2021) 10:e66788. doi: 10.7554/eLife.66788 33821796 PMC8102067

[107] Iwasaki S Hidaka H Uojima H Kubo H Adachi K Wada N . A case of successful treatment with lenvatinib in primary hepatoid adenocarcinoma of the gallbladder that was difficult to distinguish from hepatocellular carcinoma. Clin J Gastroenterol. (2022) 15:1108–14. doi: 10.1007/s12328-022-01686-6 36001231

[108] Lei L Yang L Xu YY Chen HF Zhan P Wang WX . Hepatoid adenocarcinoma of the lung: an analysis of the Surveillance, Epidemiology, and End Results (SEER) database. Open Med (Wars). (2021) 16:169–74. doi: 10.1515/med-2021-0215 33585692 PMC7863000

