# Supplementary material for: Hepatoid adenocarcinoma of the lung with normal serum alpha-fetoprotein managed with thoracic radiotherapy followed by tislelizumab plus lenvatinib: a case report and systematic review
Source: Front Oncol. 2026 Sep 18;16:1963662. doi: 10.3389/fonc.2026.1963662 (PMC13630603; doi:10.3389/fonc.2026.1963662)
Supplement: Supplementary file 1 [file Table1.docx]

**Supplementary Table S1. Full search strategies for each database.**

| **Database** | **Search Strategy** |
| --- | --- |
| PubMed | (("Lung Neoplasms"[Mesh] OR lung[tiab] OR pulmonary[tiab] OR "lung cancer*"[tiab] OR "lung neoplasm*"[tiab] OR "lung tumor*"[tiab] OR "lung tumour*"[tiab] OR NSCLC[tiab])) AND (("hepatoid adenocarcinoma"[tiab] OR "hepatoid carcinoma"[tiab] OR "hepatoid tumor*"[tiab] OR "hepatoid tumour*"[tiab] OR "hepatoid neoplasm*"[tiab])) |
| Embase | ('lung tumor'/exp OR lung:ti,ab OR pulmonary:ti,ab OR 'lung cancer*':ti,ab OR 'lung neoplasm*':ti,ab OR 'lung tumor*':ti,ab OR 'lung tumour*':ti,ab OR 'non small cell lung cancer':ti,ab) AND ('hepatoid adenocarcinoma':ti,ab OR 'hepatoid carcinoma':ti,ab OR 'hepatoid tumor*':ti,ab OR 'hepatoid tumour*':ti,ab OR 'hepatoid neoplasm*':ti,ab) |
| Web of Science | TS=("lung" OR "pulmonary" OR "lung cancer*" OR "lung neoplasm*" OR "lung tumor*" OR "lung tumour*" OR "NSCLC") AND TS=("hepatoid adenocarcinoma" OR "hepatoid carcinoma" OR "hepatoid tumor*" OR "hepatoid tumour*" OR "hepatoid neoplasm*") |
| CNKI | (SU=("Lung" OR "Lung cancer" OR "Lung neoplasms" OR "Non-small cell lung cancer")) AND (SU=("Hepatoid adenocarcinoma" OR "Hepatoid carcinoma" OR "Hepatoid tumors")) |
| Wanfang Data | (Theme:("Lung" OR "Lung cancer" OR "Lung neoplasms" OR "Non-small cell lung cancer")) AND (Theme:("Hepatoid adenocarcinoma" OR "Hepatoid carcinoma" OR "Hepatoid tumors")) |

Search period: 1 January 2000 to 4 July 2026.
