# Supplementary material for: Hepatoid adenocarcinoma of the lung with normal serum alpha-fetoprotein managed with thoracic radiotherapy followed by tislelizumab plus lenvatinib: a case report and systematic review
Source: Front Oncol. 2026 Sep 18;16:1963662. doi: 10.3389/fonc.2026.1963662 (PMC13630603; doi:10.3389/fonc.2026.1963662)
Supplement: Supplementary file 2 [file Table2.docx]

**Supplementary Table S2. Methodological quality assessment of included reports using JBI critical appraisal checklists**

| **No.** | **Study (Author, Year; Ref.)** | **Design** | **Cases** | **Checklist** | **Q1** | **Q2** | **Q3** | **Q4** | **Q5** | **Q6** | **Q7** | **Q8** | **Q9** | **Q10** | **Y/applicable** |
| --- | --- | --- | --- | --- | --- | --- | --- | --- | --- | --- | --- | --- | --- | --- | --- |
| 1 | Talat F, 2026 (6) | Case report | 1 | Case report | Y | U | Y | Y | Y | Y | U | Y | NA | NA | 6/8 |
| 2 | Kapes G, 2026 (7) | Case report | 1 | Case report | Y | Y | Y | Y | Y | Y | U | Y | NA | NA | 7/8 |
| 3 | Fernández Aceñero MJ, 2025 (8) | Case report | 1 | Case report | Y | Y | Y | Y | U | U | NA | Y | NA | NA | 5/7 |
| 4 | Zahfir I, 2025 (9) | Case report | 1 | Case report | Y | Y | Y | Y | U | U | NA | Y | NA | NA | 5/7 |
| 5 | Wang S, 2016 (10) | Case report | 1 | Case report | Y | U | Y | Y | U | U | NA | Y | NA | NA | 4/7 |
| 6 | Wang X, 2025 (11) | Case report | 1 | Case report | Y | U | Y | Y | Y | Y | U | Y | NA | NA | 6/8 |
| 7 | Khabbaza R, 2025 (12) | Case report | 1 | Case report | Y | Y | Y | Y | Y | Y | NA | Y | NA | NA | 7/7 |
| 8 | Hua YY, 2025 (13) | Case report | 1 | Case report | Y | Y | Y | Y | Y | Y | U | Y | NA | NA | 7/8 |
| 9 | Harrell D, 2025 (14) | Case report | 1 | Case report | Y | Y | Y | Y | Y | Y | U | Y | NA | NA | 7/8 |
| 10 | Mo Y-J, 2024 (15) | Case report | 1 | Case report | Y | Y | Y | Y | Y | Y | U | Y | NA | NA | 7/8 |
| 11 | Huang X, 2024 (16) | Case report | 1 | Case report | Y | Y | Y | Y | Y | Y | U | Y | NA | NA | 7/8 |
| 12 | Uruga H, 2024 (17) | Case report | 1 | Case report | Y | Y | Y | Y | Y | Y | U | Y | NA | NA | 7/8 |
| 13 | Cai C, 2024 (18) | Case report | 1 | Case report | Y | Y | Y | Y | Y | Y | U | Y | NA | NA | 7/8 |
| 14 | Wang B, 2024 (19) | Case report | 1 | Case report | Y | Y | Y | Y | Y | Y | U | Y | NA | NA | 7/8 |
| 15 | Xu K, 2023 (20) | Case report | 1 | Case report | Y | Y | Y | Y | Y | Y | U | Y | NA | NA | 7/8 |
| 16 | Sun X, 2023 (21) | Case report | 1 | Case report | Y | Y | Y | Y | Y | Y | U | Y | NA | NA | 7/8 |
| 17 | Chen K, 2024 (22) | Case report | 1 | Case report | Y | Y | Y | Y | Y | Y | U | Y | NA | NA | 7/8 |
| 18 | Huang L, 2024 (23) | Case report | 1 | Case report | Y | Y | Y | Y | Y | Y | U | Y | NA | NA | 7/8 |
| 19 | Li Y, 2023 (24) | Case report | 1 | Case report | Y | Y | Y | Y | Y | Y | U | Y | NA | NA | 7/8 |
| 20 | Bonis A, 2023 (25) | Case series | 3 | Case series | U | Y | Y | U | U | Y | Y | Y | Y | NA | 6/9 |
| 21 | Chen Z, 2022 (26) | Case report | 1 | Case report | Y | Y | Y | Y | Y | Y | U | Y | NA | NA | 7/8 |
| 22 | Sun H, 2022 (27) | Case series | 3 | Case series | U | Y | Y | U | U | Y | Y | Y | Y | NA | 6/9 |
| 23 | Chen H, 2019 (28) | Case report | 1 | Case report | Y | Y | Y | Y | Y | Y | U | Y | NA | NA | 7/8 |
| 24 | Lagos GG, 2021 (29) | Case report | 1 | Case report | Y | Y | Y | Y | Y | Y | U | Y | NA | NA | 7/8 |
| 25 | Wang W, 2020 (30) | Case report | 1 | Case report | Y | Y | Y | Y | Y | Y | U | Y | NA | NA | 7/8 |
| 26 | Yang K, 2019 (31) | Case report | 1 | Case report | Y | Y | Y | Y | Y | Y | U | Y | NA | NA | 7/8 |
| 27 | Wang C, 2019 (32) | Case report | 1 | Case report | Y | Y | Y | Y | Y | Y | U | Y | NA | NA | 7/8 |
| 28 | Basse V, 2018 (33) | Case report | 1 | Case report | Y | Y | Y | Y | Y | Y | U | Y | NA | NA | 7/8 |
| 29 | Carlinfante G, 2000 (34) | Case report | 1 | Case report | Y | Y | Y | Y | Y | Y | U | Y | NA | NA | 7/8 |
| 30 | Kitada M, 2011 (35) | Case report | 1 | Case report | Y | U | Y | Y | Y | Y | U | Y | NA | NA | 6/8 |
| 31 | Shaib W, 2014 (36) | Case report | 1 | Case report | Y | Y | Y | Y | Y | Y | U | Y | NA | NA | 7/8 |
| 32 | Gavrancic T, 2015 (37) | Case report | 1 | Case report | Y | U | Y | Y | Y | Y | U | Y | NA | NA | 6/8 |
| 33 | Ayub A, 2019 (38) | Case report | 1 | Case report | Y | Y | Y | Y | Y | Y | U | Y | NA | NA | 7/8 |
| 34 | Li J, 2019 (39) | Case report | 1 | Case report | Y | Y | Y | Y | Y | Y | U | Y | NA | NA | 7/8 |
| 35 | Laraqui Houssein Y, 2022 (40) | Case series | 2 | Case series | U | Y | Y | U | U | Y | Y | N | Y | NA | 5/9 |
| 36 | Iino K, 2003 (41) | Case report | 1 | Case report | Y | Y | Y | Y | Y | Y | U | Y | NA | NA | 7/8 |
| 37 | Alonso-Gonzalo L, 2025 (42) | Case report | 1 | Case report | Y | Y | Y | Y | Y | Y | NA | Y | NA | NA | 7/7 |
| 38 | Ivan M, 2007 (43) | Case report | 1 | Case report | Y | Y | Y | Y | Y | Y | U | Y | NA | NA | 7/8 |
| 39 | Haninger DM, 2014 (5) | Case series | 5 | Case series | U | Y | Y | U | U | Y | Y | Y | Y | NA | 6/9 |
| 40 | Feng GW, 2013 (44) | Case report | 1 | Case report | Y | U | Y | Y | Y | Y | U | Y | NA | NA | 6/8 |
| 41 | Liu HY, 2014 (45) | Case report | 1 | Case report | Y | Y | Y | Y | Y | Y | U | Y | NA | NA | 7/8 |
| 42 | Grossman K, 2014 (46) | Case report | 1 | Case report | Y | U | Y | Y | U | U | NA | Y | NA | NA | 4/7 |
| 43 | Al-Najjar H, 2015 (47) | Case report | 1 | Case report | Y | Y | Y | Y | Y | Y | U | Y | NA | NA | 7/8 |
| 44 | Matsuda Y, 2015 (48) | Case report | 1 | Case report | Y | Y | Y | Y | Y | Y | U | Y | NA | NA | 7/8 |
| 45 | Esa NYM, 2018 (49) | Case report | 1 | Case report | Y | Y | Y | Y | Y | Y | U | Y | NA | NA | 7/8 |
| 46 | Chen L, 2020 (50) | Case report | 1 | Case report | Y | Y | Y | Y | Y | Y | U | Y | NA | NA | 7/8 |
| 47 | Chen J-X, 2021 (51) | Case report | 1 | Case report | Y | U | Y | Y | Y | Y | NA | Y | NA | NA | 6/7 |
| 48 | Miyama Y, 2020 (52) | Case report | 1 | Case report | Y | Y | Y | Y | Y | Y | U | Y | NA | NA | 7/8 |
| 49 | Hou Q, 2017 (53) | Case report | 1 | Case report | Y | Y | Y | Y | Y | U | U | Y | NA | NA | 6/8 |
| 50 | Zhong MY, 2015 (54) | Case report | 1 | Case report | Y | Y | Y | Y | Y | Y | U | Y | NA | NA | 7/8 |
| 51 | He XY, 2020 (55) | Case report | 1 | Case report | Y | Y | Y | Y | Y | Y | U | Y | NA | NA | 7/8 |
| 52 | Pasricha S, 2021 (56) | Case series | 6 | Case series | U | Y | Y | U | U | Y | Y | Y | Y | NA | 6/9 |
| 53 | Meyers M, 2022 (57) | Case report | 1 | Case report | Y | Y | Y | Y | Y | Y | U | Y | NA | NA | 7/8 |
| 54 | Yao Y, 2022 (58) | Case report | 1 | Case report | Y | U | Y | Y | Y | Y | U | Y | NA | NA | 6/8 |
| 55 | Arai H, 2025 (59) | Case report | 1 | Case report | Y | Y | Y | Y | Y | Y | U | Y | NA | NA | 7/8 |
| 56 | Cao YA, 2008 (60) | Case report | 1 | Case report | Y | U | Y | Y | Y | Y | U | Y | NA | NA | 6/8 |
| 57 | Ding QJ, 2019 (61) | Case report | 1 | Case report | Y | Y | Y | Y | Y | Y | U | Y | NA | NA | 7/8 |
| 58 | Xu YJ, 2020 (62) | Case series | 2 | Case series | U | Y | Y | U | U | Y | Y | Y | Y | NA | 6/9 |
| 59 | Fan XL, 2019 (63) | Case report | 1 | Case report | Y | Y | Y | Y | Y | Y | U | Y | NA | NA | 7/8 |
| 60 | Li Y, 2019 (64) | Case report | 1 | Case report | Y | U | Y | Y | Y | Y | U | Y | NA | NA | 6/8 |
| 61 | Yu DZ, 2020 (65) | Case report | 1 | Case report | Y | U | Y | Y | Y | Y | U | Y | NA | NA | 6/8 |
| 62 | Shen C, 2021 (66) | Case report | 1 | Case report | Y | Y | Y | Y | Y | Y | U | Y | NA | NA | 7/8 |
| 63 | Zeng ZY, 2023 (67) | Case report | 1 | Case report | Y | Y | Y | Y | Y | Y | U | Y | NA | NA | 7/8 |
| 64 | Cao DM, 2023 (68) | Case series | 3 | Case series | U | Y | Y | U | U | Y | Y | N | Y | NA | 5/9 |
| 65 | Liu GR, 2023 (69) | Case report | 1 | Case report | Y | Y | Y | Y | Y | Y | U | Y | NA | NA | 7/8 |
| 66 | Zhang K, 2023 (70) | Case report | 1 | Case report | Y | Y | Y | Y | Y | Y | NA | Y | NA | NA | 7/7 |
| 67 | Zhang YY, 2023 (71) | Case report | 1 | Case report | Y | Y | Y | Y | U | U | NA | Y | NA | NA | 5/7 |
| 68 | Li Q, 2023 (72) | Case report | 1 | Case report | Y | Y | Y | Y | Y | Y | U | Y | NA | NA | 7/8 |
| 69 | Zou LQ, 2023 (73) | Case series | 2 | Case series | U | Y | Y | U | U | Y | Y | Y | Y | NA | 6/9 |
| 70 | Hong JD, 2025 (74) | Case report | 1 | Case report | Y | Y | Y | Y | Y | Y | U | Y | NA | NA | 7/8 |
| 71 | Bai CG, 2006 (75) | Case report | 1 | Case report | Y | U | Y | Y | Y | Y | U | Y | NA | NA | 6/8 |
| 72 | Seddon JE, 2021 (76) | Case report | 1 | Case report | Y | Y | Y | Y | Y | Y | U | Y | NA | NA | 7/8 |
| 73 | Shahrudin MS, 2023 (77) | Case report | 1 | Case report | Y | U | Y | Y | Y | U | U | Y | NA | NA | 5/8 |
| 74 | Cavalcante LB, 2013 (78) | Case report | 1 | Case report | Y | Y | Y | Y | Y | Y | NA | Y | NA | NA | 7/7 |
| 75 | Shao Y, 2016 (79) | Case report | 1 | Case report | Y | U | Y | Y | Y | U | U | Y | NA | NA | 5/8 |
| 76 | Lucas C, 2018 (80) | Case report | 1 | Case report | Y | Y | Y | Y | Y | Y | NA | Y | NA | NA | 7/7 |
| 77 | Ruiz Cerrato D, 2018 (81) | Case report | 1 | Case report | Y | Y | Y | Y | Y | Y | U | Y | NA | NA | 7/8 |
| 78 | Terracciano LM, 2003 (82) | Case report | 1 | Case report | Y | U | Y | Y | Y | Y | U | Y | NA | NA | 6/8 |
| 79 | Genova S, 2003 (83) | Case report | 1 | Case report | Y | U | Y | Y | Y | Y | U | Y | NA | NA | 6/8 |
| 80 | Kuan K, 2019 (84) | Case report | 1 | Case report | Y | Y | Y | Y | Y | Y | U | Y | NA | NA | 7/8 |
| 81 | Hayashi Y, 2002 (85) | Case report | 1 | Case report | Y | Y | Y | Y | Y | Y | U | Y | NA | NA | 7/8 |
| 82 | No EJ, 2006 (86) | Case report | 1 | Case report | Y | Y | Y | Y | Y | Y | U | Y | NA | NA | 7/8 |
| 83 | Wang PC, 2008 (87) | Case report | 1 | Case report | Y | Y | Y | Y | Y | Y | U | Y | NA | NA | 7/8 |
| 84 | Papatsimpas G, 2012 (88) | Case report | 1 | Case report | Y | U | Y | Y | Y | Y | U | Y | NA | NA | 6/8 |
| 85 | Mokrim M, 2012 (89) | Case report | 1 | Case report | Y | Y | Y | Y | Y | Y | U | Y | NA | NA | 7/8 |
| 86 | Che YQ, 2014 (90) | Case report | 1 | Case report | Y | Y | Y | Y | Y | Y | U | Y | NA | NA | 7/8 |
| 87 | Sun JN, 2016 (91) | Case report | 1 | Case report | Y | Y | Y | Y | Y | Y | U | Y | NA | NA | 7/8 |
| 88 | Nakashima K, 2018 (92) | Case report | 1 | Case report | Y | U | Y | Y | Y | Y | U | Y | NA | NA | 6/8 |
| 89 | Shi YF, 2019 (93) | Case report | 1 | Case report | Y | Y | Y | Y | Y | Y | U | Y | NA | NA | 7/8 |
| 90 | El Khoury A, 2019 (94) | Case report | 1 | Case report | Y | Y | Y | Y | Y | Y | U | Y | NA | NA | 7/8 |
| 91 | Cancela Díez B, 2019 (95) | Case report | 1 | Case report | Y | Y | U | Y | Y | Y | U | Y | NA | NA | 6/8 |

**Abbreviations:** Y = Yes; N = No; U = Unclear; NA = Not applicable.

**JBI case report domains:** Q1, demographic characteristics; Q2, patient history and timeline; Q3, clinical condition on presentation; Q4, diagnostic tests or assessment methods and results; Q5, intervention or treatment; Q6, post-intervention clinical condition or follow-up; Q7, adverse or unanticipated events; Q8, takeaway lessons.

**JBI case series domains:** Q1, clear inclusion criteria; Q2, standard and reliable measurement of the condition; Q3, valid methods for identifying the condition; Q4, consecutive inclusion of participants; Q5, complete inclusion of participants; Q6, demographic reporting; Q7, clinical information reporting; Q8, outcomes or follow-up reporting; Q9, reporting of presenting site(s) or clinic(s); Q10, appropriate statistical analysis.

**Note:** The final column reports the number of “Yes” responses among applicable items and is presented descriptively without applying a quality cutoff.
