# Supplementary material for: Hepatoid adenocarcinoma of the lung with normal serum alpha-fetoprotein managed with thoracic radiotherapy followed by tislelizumab plus lenvatinib: a case report and systematic review
Source: Front Oncol. 2026 Sep 18;16:1963662. doi: 10.3389/fonc.2026.1963662 (PMC13630603; doi:10.3389/fonc.2026.1963662)
Supplement: Supplementary file 3 [file Table3.docx]

**Supplementary Table S3. Clinicopathologic characteristics and serum markers of included cases with primary hepatoid adenocarcinoma of the lung (HAL).**

| **No.** | Study (Author, Year; Ref.) | **Country** | **Age** | **Sex** | **Smoking history*** | **Primary location** | **Tumor size (cm)** | **Nodal/distant involvement **** | **TNM stage***** | **Serum**  **AFP** | **Serum**  **CEA** |
| --- | --- | --- | --- | --- | --- | --- | --- | --- | --- | --- | --- |
| 1 | Talat F, 2026 (6) | USA | 82 | M | NA | RLL | NA | None | cT3N0M0 (IIB) | NA | NA |
| 2 | Kapes G, 2026 (7) | Greece | 64 | M | 50 | LUL | 4.5 × 4.0 × 3.5 | Mandible, Hilar LN | cT2bN1M1b (IVA) | Normal | NA |
| 3 | Fernández Aceñero MJ, 2025 (8) | Spain | 67 | M | 23 | RLL | 4.3 | Bone, Hilar LN, Mediastinal LN, Cervical LN | cT2bN3M1c (IVB) | NA | NA |
| 4 | Zahfir I, 2025 (9) | Morocco | 39 | M | Yes | Right lung | 15.6 × 15.3 × 16.2 | NA | cT4NxM0 (stage group NA) | ↑ | NA |
| 5 | Wang S, 2016 (10) | China | 56 | M | NA | RUL | 4.8 | Hilar LN | cT2bN1M0 (IIB) | NA | NA |
| 6 | Wang X, 2025 (11) | China | 66 | M | NA | LLL | 0.8–2.0 | Contralateral lung, Hilar LN | cT4N1M1a (IVA) | ↑ | NA |
| 7 | Khabbaza R, 2025 (12) | Syria | 49 | M | 20 | Left lung | NA | Skin, Liver, Bone | cTxNxM1c (IVB) | Normal | NA |
| 8 | Hua YY, 2025 (13) | China | 45 | F | No | RLL | 6.0 × 4.4 | Bone | cT3N0M1b (IVA) | ↑ | Normal |
| 9 | Harrell D, 2025 (14) | USA | 47 | F | Yes | RUL | 11.0 × 6.4 × 8.2 | NA | cT4NxM0 (Stage III, subgroup NA) | ↑ | NA |
| 10 | Mo Y-J, 2024 (15) | China | 76 | M | 360 | RML | 7.1 × 4.7 | Bone, Mediastinal LN, Adrenal | cT4N2M1c (IVB) | Normal | NA |
| 11 | Huang X, 2024 (16) | China | 47 | M | 30 | RUL | 10.0 × 7.5 | None | cT4N0M0 (IIIA) | Normal | NA |
| 12 | Uruga H, 2024 (17) | Japan | 70s† | M | 62.5 | RML | 1.9 | None | pT1bN0M0 (IA2) | NA | Normal |
| 13 | Cai C, 2024 (18) | China | 75 | F | 30 | RUL | 10.7 | Mediastinal LN | cT4N3M0 (IIIC) | ↑ | ↑ |
| 14 | Wang B, 2024 (19) | China | 69 | M | 50 | LLL | 6.0 × 5.0 × 3.2 | Hilar LN | pT3N1Mx (stage group NA) | NA | NA |
| 15 | Xu K, 2023 (20) | China | 65 | M | 40 | LUL | 3.0 × 2.8 | Mediastinal LN, Hilar LN, Axillary LN | cT1cN2M1b (IVA) | ↑ | ↑ |
| 16 | Sun X, 2023 (21) | China | 46 | M | 45 | LUL | 9.1 × 6.1 | None | cT3N0M0 (IIB) | ↑ | ↑ |
| 17 | Chen K, 2024 (22) | China | 58 | M | Yes | RLL | 8.6 × 6.4 | Hilar LN, Mediastinal LN | cT4N2M0 (IIIB) | ↑ | NA |
| 18 | Huang L, 2024 (23) | China | 59 | M | 30 | RLL | 3.5 × 3.3 | Mediastinal LN | pT2bN2M0 (IIIA) | Normal | NA |
| 19 | Li Y, 2023 (24) | China | 64 | M | 40 | RUL | NA | Brain, Mediastinal LN, Hilar LN, Lung | Stage IV (exact M category uncertain) | ↑ | ↑ |
| 20 | Bonis A, 2023 (25) | Italy | 67 | F | No | LUL | 6.8 | Bone, Buttock | cT3NxM1c (IVB) | NA | NA |
| 21 | Bonis A, 2023 (25) | Italy | 61 | F | Yes | RUL | 6.5 × 5.7 | Mediastinal LN | cT3N2M0 (IIIB) | NA | NA |
| 22 | Bonis A, 2023 (25) | Italy | 78 | M | Yes | LLL | 3.3 | Mediastinal LN | pT1bN3M0 (IIIB) | NA | NA |
| 23 | Chen Z, 2022 (26) | China | 67 | M | 90 | RML | 7.1 × 5.3 | Hilar LN, Mediastinal LN | cT4N2M0 (IIIB) | Normal | Normal |
| 24 | Sun H, 2022 (27) | China | 66 | M | Yes | RUL | 3.2 × 2.5 × 2.0 | None | pT2aN0M0 (IB) | NA | NA |
| 25 | Sun H, 2022 (27) | China | 64 | M | No | LLL | 4.0 × 3.0 | None | pT2aN0M0 (IB) | NA | NA |
| 26 | Sun H, 2022 (27) | China | 64 | M | No | LUL | 8.0 × 7.5 × 4.5 | Hilar LN | pT4N1M0 (IIIA) | ↑ | NA |
| 27 | Chen H, 2019 (28) | China | 53 | M | No | RUL | 5.3 × 3.5 | None | pT3N0M0 (IIB) | ↑ | Normal |
| 28 | Lagos GG, 2021 (29) | USA | 54 | M | 25 | RLL | 14.0 | Pleura, Mediastinal LN, Hilar LN, Bone, Adrenal | cT4NxM1c (IVB) | ↑ | NA |
| 29 | Wang W, 2020 (30) | China | 41 | M | 180 | RUL | 5.2 × 5.8 | Hilar LN, Supraclavicular LN | cT3N3M0 (IIIC) | Normal | Normal |
| 30 | Yang K, 2019 (31) | China | 70 | M | 120 | LLL | 6.0 × 6.0 × 5.5 | Hilar LN | pT3N1M0 (IIIA) | Normal | ↑ |
| 31 | Wang C, 2019 (32) | China | 70 | M | 50 | RUL | 6.0 × 4.6 | Hilar LN, Mediastinal LN | cT3N2M0 (IIIB) | Normal | NA |
| 32 | Basse V, 2018 (33) | France | 66 | F | 40 | LLL | 6.3 | LN (site unspecified), Brain, Adrenal, Bone | cTxNxM1c (IVB) | ↑ | Normal |
| 33 | Carlinfante G, 2000 (34) | Italy | 63 | M | Yes | LUL | 15.0 × 11.0 × 8.0 | Adrenal, Bone | cT4NxM1c (IVB) | ↑ | NA |
| 34 | Kitada M, 2011 (35) | Japan | 69 | M | NA | RLL | 7.0 | Hilar LN | pT3N1M0 (IIIA) | ↑ | ↑ |
| 35 | Shaib W, 2014 (36) | USA | 53 | F | 40 | RUL | 9.5 × 9.0 × 8.0 | None | pT4N0M0 (IIIA) | ↑ | NA |
| 36 | Gavrancic T, 2015 (37) | USA | 64 | M | NA | RUL | 3.8 × 2.9 | Hilar LN, Mediastinal LN, Bone | cT2aN2M1c (IVB) | ↑ | NA |
| 37 | Ayub A, 2019 (38) | USA | 61 | M | 40 | RUL | 2.3 | None | pT1cN0M0 (IA3) | NA | NA |
| 38 | Li J, 2019 (39) | China | 71 | M | No | RLL | 7.0 × 4.5 | Hilar LN, Mediastinal LN, Supraclavicular LN, Bone | cT4N3M1b (IVA) | ↑ | NA |
| 39 | Laraqui Houssein Y, 2022 (40) | Morocco | 70 | M | 37 | LUL | 5.0 | None | pT2bN0M0 (IIA) | Normal | NA |
| 40 | Laraqui Houssein Y, 2022 (40) | Morocco | 70 | M | Yes | Right lung | NA | NA | Stage group NA (exact TNM NA) | Normal | NA |
| 41 | Iino K, 2003 (41) | Japan | 63 | M | 200 | RUL | 2.8 × 2.6 × 2.4 | None | pT1cN0M0 (IA3) | ↑ | Normal |
| 42 | Alonso-Gonzalo L, 2025 (42) | Spain | 72 | M | Yes | Left lung | NA | Multiple liver | cTxNxM1c (IVB) | Normal | NA |
| 43 | Ivan M, 2007 (43) | Canada | 54 | M | 40 | LUL | 13.0 × 11.0 | Mediastinal LN, Brain | cT4N2M1b (IVA) | ↑ | NA |
| 44 | Haninger DM, 2014 (5) | USA | 51 | M | 45 | Right lung | 4.3 × 3.0 | Mediastinal LN, Supraclavicular LN | cT2bN3M0 (IIIB) | Normal | NA |
| 45 | Haninger DM, 2014 (5) | USA | 52 | M | 40 | RUL | 2.5 | Adrenal, Brain | cT1cN0M1c (IVB) | NA | NA |
| 46 | Haninger DM, 2014 (5) | USA | 64 | M | 75 | LUL | 3.2 × 2.2 | Bone | cT2aN0M1b (IVA) | Normal | NA |
| 47 | Haninger DM, 2014 (5) | USA | 54 | F | 35 | LUL | 1.0 | Bone | cT1aN0M1b (IVA) | NA | NA |
| 48 | Haninger DM, 2014 (5) | USA | 60 | M | 40 | RUL | 11.2 × 10.1 × 8.5 | Brain, Hilar LN, Mediastinal LN | cT4N2M1b (IVA) | ↑ | NA |
| 49 | Feng GW, 2013 (44) | China | 46 | M | NA | LUL | 2.0 × 2.4 | None | pT1cN0M0 (IA3) | ↑ | Normal |
| 50 | Liu HY, 2014 (45) | China | 64 | M | Yes | RLL | 8.0 × 5.5 × 5.0 | None | pT4N0M0 (IIIA) | ↑ | Normal |
| 51 | Grossman K, 2014 (46) | USA | 54 | M | NA | Right lung | 4.0 × 5.0 | Brain | cT2bNxM1b (IVA) | Normal | NA |
| 52 | Al-Najjar H, 2015 (47) | UK | 71 | M | 80 | Right lung | 3.0 | Contralateral lung, Mediastinal LN | cTxN2M1a (IVA) | ↑ | NA |
| 53 | Matsuda Y, 2015 (48) | Japan | 75 | M | 55 | LLL | 2.2 × 1.5 | None | pT1cN0M0 (IA3) | ↑ | ↑ |
| 54 | Esa NYM, 2018 (49) | Malaysia | 50 | M | 40 | LUL | 7.2 × 7.4 × 8.6 | None | cT4N0M0 (IIIA) | ↑ | Normal |
| 55 | Chen L, 2020 (50) | China | 65 | F | No | RLL | 3.7 × 3.6 | Contralateral lung, Bone | Stage IV (exact TNM NA) | ↑ | ↑ |
| 56 | Chen J-X, 2021 (51) | China | 63 | M | NA | LUL | NA | LN (site unspecified), Multiple subcutaneous | cTxNxM1c (IVB) | NA | ↑ |
| 57 | Miyama Y, 2020 (52) | Japan | 58 | M | 31 | RLL | 7.5 | Pleura, Contralateral lung, Bone, Brain | cT3/4NxM1c (IVB) | NA | ↑ |
| 58 | Hou Q, 2017 (53) | China | 59 | M | Yes | RUL | 4.5 × 3.5 × 3.5 | None | pT2bN0M0 (IIA) | Normal | Normal |
| 59 | Zhong MY, 2015 (54) | China | 61 | M | 40 | LUL | 5.7 × 4.3 | Bone | cT3NxM1c (IVB) | ↑ | ↑ |
| 60 | He XY, 2020 (55) | China | 71 | M | 50 | RLL | 6.9 × 5.4 | Mediastinal LN | cT3N3M0 (IIIC) | ↑ | ↑ |
| 61 | Pasricha S, 2021 (56) | India | 40 | M | Yes | LUL | 4.4 | Mediastinal LN | Stage IIIB (exact TNM NA) | ↑ | NA |
| 62 | Pasricha S, 2021 (56) | India | 74 | M | Yes | LUL | 5.9 | Mediastinal LN, Brain | Stage IV (exact TNM NA) | Normal | NA |
| 63 | Pasricha S, 2021 (56) | India | 52 | M | No | RUL | 6.0 | Mediastinal LN, Pleura | cT3N2M1a (IVA) | Normal | NA |
| 64 | Pasricha S, 2021 (56) | India | 65 | M | Yes | Right lung | 4.0 | Cervical LN, Mediastinal LN, Pleura | Stage IV (exact TNM NA) | Normal | NA |
| 65 | Pasricha S, 2021 (56) | India | 67 | M | Yes | RUL | 5.5 | Cervical LN, Mediastinal LN | Stage IV (exact TNM NA) | NA | NA |
| 66 | Pasricha S, 2021 (56) | India | 26 | M | No | Lung, site unspecified | 8.0 | Contralateral lung, Mediastinal LN, Liver | Stage IV / cTxN2M1c (IVB) | ↑ | NA |
| 67 | Meyers M, 2022 (57) | USA | 69 | M | Yes | Lung, site unspecified | 3.1 | Mediastinal LN, Liver | cT2aN2M1b (IVA) | NA | NA |
| 68 | Yao Y, 2022 (58) | China | 63 | M | NA | LUL | 7.5 × 5.5 | None | pT4N0M0 (IIIA) | Normal | ↑ |
| 69 | Arai H, 2025 (59) | Japan | 55 | M | 30 | LLL | 7.3 | Hilar LN, Liver, Bone | cT4N1M1c (IVB) | Normal | ↑ |
| 70 | Cao YA, 2008 (60) | China | 64 | M | NA | RUL | 3.0 × 2.0 × 1.4 | Multiple liver | cT1cN0M1c (IVB) | ↑ | NA |
| 71 | Ding QJ, 2019 (61) | China | 52 | M | 30 | RUL | 7.0 × 6.0 × 4.5 | Mediastinal LN | pT3N2M0 (IIIB) | ↑ | Normal |
| 72 | Xu YJ, 2020 (62) | China | 65 | M | 40 | RUL | 5.89 × 5.34 | Bone | cT3NxM1c (IVB) | Normal | NA |
| 73 | Xu YJ, 2020 (62) | China | 71 | M | 40 | RUL | 5.5 × 4.4 | Supraclavicular LN, Mediastinal LN, Hilar LN | cT3N3M0 (IIIC) | Normal | NA |
| 74 | Fan XL, 2019 (63) | China | 70 | M | 120 | LLL | 6.0 × 6.0 × 1.5 | Hilar LN | pT3N1M0 (IIIA) | NA | ↑ |
| 75 | Li Y, 2019 (64) | China | 74 | M | NA | RUL | NA | Mediastinal LN, Hilar LN, Adrenal, Bone | cTxN3M1c (IVB) | Normal | ↑ |
| 76 | Yu DZ, 2020 (65) | China | 47 | M | NA | LLL | 8.0 × 6.0 × 5.0 | None | pT4N0M0 (IIIA) | ↑ | NA |
| 77 | Shen C, 2021 (66) | China | 66 | M | 37.5 | RUL | 4.1 × 4.0 × 3.5 | None | pT2bN0M0 (IIA) | ↑ | Normal |
| 78 | Zeng ZY, 2023 (67) | China | 49 | M | 60 | Right lung | 5.0 × 4.0 | Mediastinal LN, Supraclavicular LN | cTxN3M0 (stage III, exact T/stage NA) | Normal | ↑ |
| 79 | Cao DM, 2023 (68) | China | 58 | M | Yes | RUL | 1.13 × 1.57 | Brain | cT1bNxM1b (IVA) | Normal | NA |
| 80 | Cao DM, 2023 (68) | China | 62 | M | No | Lung, site unspecified | 5.50× 3.84 | Contralateral lung | cT3NxM1a (IVA) | Normal | NA |
| 81 | Cao DM, 2023 (68) | China | 66 | M | No | RUL | 5.83 × 7.19 | NA | cT4NxM0 (Stage III, subgroup NA) | Normal | NA |
| 82 | Liu GR, 2023 (69) | China | 83 | M | 50 | Left lung | 4.4 × 4.0 | Supraclavicular LN, Mediastinal LN, Retroperitoneal LN, Inguinal LN, Adrenal | cT2bN3M1c (IVB) | ↑ | ↑ |
| 83 | Zhang K, 2023 (70) | China | 83 | M | 60 | RLL | 5.0 × 5.8 × 6.8 | Mediastinal LN, Hilar LN, Pleura | cT4N3M1a (IVA) | ↑ | ↑ |
| 84 | Zhang YY, 2023 (71) | China | 61 | M | 40 | Left hilar/paramediastinal lung | NA | Mediastinal LN, Pleura | cT4N2M1a (IVA), exact TNM uncertain | ↑ | ↑ |
| 85 | Li Q, 2023 (72) | China | 65 | M | 20 | Right lung | 14.0 × 10.7 × 9.5 | Supraclavicular LN, Mediastinal LN, Hilar LN, Pleura, Adrenal, Brain | cT4N3M1c (IVB) | ↑ | NA |
| 86 | Zou LQ, 2023 (73) | China | 64 | M | No | LUL | 7.0 × 4.7 × 3.6 | Bone | cT3NxM1b (IVA) | NA | NA |
| 87 | Zou LQ, 2023 (73) | China | 47 | M | 20 | LLL | 5.3 × 4.0 × 3.9 | None | pT3N0M0 (IIB) | NA | NA |
| 88 | Hong JD, 2025 (74) | China | 59 | M | 30 | RLL | 4.0 × 3.0 × 7.4 | Mediastinal LN | cT2N2M0 (IIIA) | ↑ | NA |
| 89 | Bai CG, 2006 (75) | China | 48 | M | NA | RUL | 10.0 × 8.0 × 6.0 | NA | **pT4NxM0 (Stage III, subgroup NA)** | ↑ | Normal |
| 90 | Seddon JE, 2021 (76) | USA | 33 | M | Yes | Left lung | NA | Mediastinal LN, Brain, Bone, Contralateral lung | cT2N2M1c (IVB) | NA | NA |
| 91 | Shahrudin MS, 2023 (77) | Malaysia | 71 | M | NA | RUL | NA | Liver, Gallbladder, Bone, Hard palate | cTxNxM1c (IVB) | NA | NA |
| 92 | Cavalcante LB, 2013 (78) | Brazil | 66 | M | Yes | RLL | NA | Adrenal, Mediastinal LN | cTxN2M1b (IVA) | NA | NA |
| 93 | Shao Y, 2016 (79) | China | 42 | M | NA | Right lung | 12.4 | NA | Stage IIIB (exact TNM NA) | NA | NA |
| 94 | Lucas C, 2018 (80) | Portugal | 80 | M | 60 | Right lung | NA | Mediastinal LN, Hilar LN, Bone | cTxN2M1b (IVA) | NA | NA |
| 95 | Ruiz Cerrato D, 2018 (81) | USA | 69 | F | 70 | LUL | 5.7 × 4.3 × 5.7 | Mediastinal LN, Hilar LN | cT3N2M0 (IIIB) | Normal | NA |
| 96 | Terracciano LM, 2003 (82) | Switzerland | 49 | M | NA | LLL | 5.0 | Liver, Adrenal, Brain, Hilar LN | cTxN1M1c (IVB) | ↑ | NA |
| 97 | Genova S, 2003 (83) | Bulgaria | 71 | M | NA | LUL | 7.7 × 6.4 | None | pT4N0M0 (IIIA) | NA | NA |
| 98 | Kuan K, 2019 (84) | USA | 47 | M | Yes | RUL | 14.0 | NA | pT4NxM0 (Stage III, subgroup NA) | NA | NA |
| 99 | Hayashi Y, 2002 (85) | Japan | 55 | M | 87.5 | RUL | 5.0 × 4.8 × 6.5 | None | pT3N0M0 (IIB) | Normal | ↑ |
| 100 | No EJ, 2006 (86) | Korea | 64 | M | Yes (40 yr) | RUL | 9.0 × 8.0 | Hilar LN | cT4N1M0 (IIIA) | ↑ | ↑ |
| 101 | Wang PC, 2008 (87) | China | 44 | M | 40 | RUL | 10.0 | Mediastinal LN | cT4N2M0 (IIIB) | ↑ | NA |
| 102 | Papatsimpas G, 2012 (88) | Greece | 48 | M | NA | RUL | 20.0 × 11.0 × 8.0 | Mediastinal LN | cT4N2M0 (IIIB) | ↑ | NA |
| 103 | Mokrim M, 2012 (89) | Morocco | 52 | M | 20 | LUL | 11.8 × 12.0 × 8.0 | Hilar LN | cT4N1M0 (IIIA) | ↑ | NA |
| 104 | Che YQ, 2014 (90) | China | 48 | M | 70 | Left lung | 7.9 × 10.0 | Mediastinal LN | Stage IIIA (N2; exact TNM NA) | ↑ | Normal |
| 105 | Sun JN, 2016 (91) | China | 59 | M | Yes (ceased 9 yr) | RUL | 4.5 × 3.5 × 3.5 | None | pT2bN0M0 (IIA) | Normal | Normal |
| 106 | Nakashima K, 2018 (92) | Japan | 60 | M | NA | RUL | 6.3 × 4.8 | None | pT3N0M0 (IIB) | ↑ | ↑ |
| 107 | Shi YF, 2019 (93) | China | 60 | M | No | RUL | 7.0 × 7.0 × 5.0 | Mediastinal LN | pT3N2M0 (IIIB) | ↑ | Normal |
| 108 | El Khoury A, 2019 (94) | UK | 59 | M | > 30 | RUL | 9.3 × 7.2 × 6.8 | Liver, Mediastinal LN | cT4N2M1b (IVA) | Normal | ↑ |
| 109 | Cancela Díez B, 2019 (95) | NA | 63 | M | Yes | Lung, site unspecified | NA | Skin | Stage IV (exact TNM NA) | NA | NA |
| **Summary** |  | **China:** 54/108 (50%);  **USA:** 16/108 (15%); **Japan:** 8/108 (7%);  **India:** 6/108 (6%);  **Others:** 24/108 **(22%)** | **Median** 63 (IQR: 53–67; range: 26–83; n=109) | **M: 99/109 (91%);**  **F: 10/109 (9%)** | **Yes:** 77/90 (86%);  **No:** 13/90 (14%);  **NA:** 19 | **Right lung:** 66/105 (63%);  **Left lung:** 39/105 (37%);  **most common lobe:** RUL, 38/89 (43%)  **Site unspecified/indeterminate:** 4 | **Median maximum diameter:** 6 cm (IQR: 4.4–8; range: 1–20; n=96);  **NA:** 13 | **None:** 24/102 (24%); **regional nodal involvement:** 57/102 (56%);  **distant metastasis:** 50/102 (49%); **common distant sites:** bone 22/102 (22%), brain 12/102 (12%), adrenal 10/102 (10%), liver 9/102 (9%), pleura 7/102 (7%), contralateral lung 7/102 (7%);  **NA:** 7 | **Stage I:** 7/106 (7%)**; Stage II:** 11/106 (10%); **Stage III:** 38/106 (36%); **Stage IV:** 50/106 (47%); **stage group NA:** 3 | **↑: 4**9/81 (60%); **Normal:** 32/81 (40%); **NA:** 28 | **↑:** 24/41 (59%); **Normal:** 17/41 (41%);  **NA:** 68 |

**Notes:** * Smoking history was recorded as pack-years when available; Yes indicates smoking history without reported pack-years, and No indicates no smoking history.

** Nodal and distant involvement refers to findings at initial diagnosis. ***TNM stage was extracted as reported; if unavailable, it was assigned per AJCC eighth edition.

† Age range as reported.

**Abbreviations:** **AFP**, alpha-fetoprotein; **CEA**, carcinoembryonic antigen; **F**, female; **LN**, lymph node; **M**, male; **NA**, not available; ↑, elevated; **LLL**, left lower lobe; **LUL**, left upper lobe; **RLL**, right lower lobe; **RML**, right middle lobe; **RUL**, right upper lobe.
