# Supplementary material for: Hepatoid adenocarcinoma of the lung with normal serum alpha-fetoprotein managed with thoracic radiotherapy followed by tislelizumab plus lenvatinib: a case report and systematic review
Source: Front Oncol. 2026 Sep 18;16:1963662. doi: 10.3389/fonc.2026.1963662 (PMC13630603; doi:10.3389/fonc.2026.1963662)
Supplement: Supplementary file 4 [file Table4.docx]

**Supplementary Table S4. Pathological features, molecular profiles, treatment categories, and outcomes of included cases with primary hepatoid adenocarcinoma of the lung (HAL).**

| **No.** | **Study (Author, Year; Ref.)** | **TTF-1** | **PD-L1** | **Molecular profile*** | **Treatment category** | **OS (Status**)** |
| --- | --- | --- | --- | --- | --- | --- |
| 1 | Talat F, 2026 (6) | NA | NA | WT | Chemo + IO | 6 m (Deceased) |
| 2 | Kapes G, 2026 (7) | + | NA | NA | Surg + Chemo + RT | 6 m (Deceased) |
| 3 | Fernández Aceñero MJ, 2025 (8) | + | <1% | WT | NA | NA |
| 4 | Zahfir I, 2025 (9) | + | NA | NA | NA | NA |
| 5 | Wang S, 2016 (10) | NA | NA | NA | NA | NA |
| 6 | Wang X, 2025 (11) | - | NA | WT | Chemo + antiangiogenic therapy + IO | >75 m (Alive) |
| 7 | Khabbaza R, 2025 (12) | - | NA | NA | Untreated | 1 m (Deceased) |
| 8 | Hua YY, 2025 (13) | - | 5% | WT | Chemo + IO + antiangiogenic therapy + RT + Surg | >34 m (Alive) |
| 9 | Harrell D, 2025 (14) | NA | NA | NA | RT + IO | NA |
| 10 | Mo Y-J, 2024 (15) | - | 2% | KRAS A146T mut | IO | 3 m (Deceased) |
| 11 | Huang X, 2024 (16) | - | NA | NA | Neoadjuvant Chemo + IO + Surg + adjuvant Chemo + IO | >60 m (Alive) |
| 12 | Uruga H, 2024 (17) | + | NA | KRAS G12C mut | Surg + Chemo + RT + IO + targeted therapy | 7 m (Deceased) |
| 13 | Cai C, 2024 (18) | - | NA | NA | Chemo | 1 m (Deceased) |
| 14 | Wang B, 2024 (19) | - | 95% | NA | Surg | NA |
| 15 | Xu K, 2023 (20) | - | 50% | WT | Surg + adjuvant Chemo + IO | >6 m (Alive) |
| 16 | Sun X, 2023 (21) | + | NA | WT | Neoadjuvant Chemo + IO + antiangiogenic therapy + Surg | NA |
| 17 | Chen K, 2024 (22) | - | 8% | TP53 mut, MAP2K1 mut | Neoadjuvant Chemo + IO + antiangiogenic therapy + Surg + adjuvant Chemo + IO | >12 m (Alive) |
| 18 | Huang L, 2024 (23) | - | <1% | STK11 mut, TP53 mut, FANCI mut, FLT4 mut, NOTCH1 mut | Surg | 19 m (Deceased) |
| 19 | Li Y, 2023 (24) | - | NA | WT | RT + Chemo + IO | 9 m (Deceased) |
| 20 | Bonis A, 2023 (25) | - | 3% | WT | Chemo + IO | 15 m (Deceased) |
| 21 | Bonis A, 2023 (25) | - | <1% | KIT mut | Chemo + RT + Surg | >5 m (Alive) |
| 22 | Bonis A, 2023 (25) | - | 90% | WT | Surg + adjuvant IO | >5 m (Alive) |
| 23 | Chen Z, 2022 (26) | + | NA | NA | Neoadjuvant Chemo + Surg + adjuvant Chemo | 13 m (Deceased) |
| 24 | Sun H, 2022 (27) | - | NA | TP53 mut, SMARCA4 mut | Surg | >46 m (Alive) |
| 25 | Sun H, 2022 (27) | - | NA | TP53 mut, CDKN2A mut, STK11 mut | Surg | 26 m (Deceased) |
| 26 | Sun H, 2022 (27) | - | NA | TP53 mut, SMARCA4 mut | Surg | >23 m (Alive) |
| 27 | Chen H, 2019 (28) | - | NA | EGFR mut, TP53 mut | Surg + adjuvant Chemo + targeted therapy + RT + antiangiogenic therapy | >29 m (Alive) |
| 28 | Lagos GG, 2021 (29) | - | <5% | CDKN2A mut, MCL1 amp, SMARCA4 mut, STAG2 mut, STK11 mut, TP53 mut, TSC1 mut | Chemo + IO | >7 m (Alive) |
| 29 | Wang W, 2020 (30) | - | NA | WT | RT | 12 m (Deceased) |
| 30 | Yang K, 2019 (31) | - | NA | WT | Surg | 18 m (Deceased) |
| 31 | Wang C, 2019 (32) | - | NA | TP53 mut | Targeted therapy + Chemo + RT + antiangiogenic therapy | 9 m (Deceased) |
| 32 | Basse V, 2018 (33) | - | <1% | MET amp | Chemo + IO | NA |
| 33 | Carlinfante G, 2000 (34) | NA | NA | NA | Chemo | 6 m (Deceased) |
| 34 | Kitada M, 2011 (35) | NA | NA | NA | Surg | NA |
| 35 | Shaib W, 2014 (36) | + | NA | NA | Surg + adjuvant Chemo | >48 m (Alive) |
| 36 | Gavrancic T, 2015 (37) | + | NA | WT | Chemo + antiangiogenic therapy + RT | 11 m (Deceased) |
| 37 | Ayub A, 2019 (38) | + | NA | NA | Surg + RT | 6 m (Deceased) |
| 38 | Li J, 2019 (39) | - | <1% | FAT1 mut, FAT1 CN loss | RT + antiangiogenic therapy | 4 m (Deceased) |
| 39 | Laraqui Houssein Y, 2022 (40) | + | NA | NA | Surg | NA |
| 40 | Laraqui Houssein Y, 2022 (40) | + | NA | NA | Surg | NA |
| 41 | Iino K, 2003 (41) | NA | NA | NA | Surg | >5 m (Alive) |
| 42 | Alonso-Gonzalo L, 2025 (42) | - | NA | NA | BSC | 1 m (Deceased) |
| 43 | Ivan M, 2007 (43) | + | NA | NA | Chemo + RT | NA |
| 44 | Haninger DM, 2014 (5) | + | NA | NA | Chemo + RT | 14 m (Deceased) |
| 45 | Haninger DM, 2014 (5) | + | NA | NA | Surg + Chemo + RT | >37 m (Alive) |
| 46 | Haninger DM, 2014 (5) | + | NA | WT | Surg + Chemo + RT | 10 m (Deceased) |
| 47 | Haninger DM, 2014 (5) | + | NA | NA | Chemo + RT + Surg | >108 m (Alive) |
| 48 | Haninger DM, 2014 (5) | + | NA | NA | Chemo + RT | >1 m (Alive) |
| 49 | Feng GW, 2013 (44) | + | NA | NA | Surg + adjuvant Chemo | >6 m (Alive) |
| 50 | Liu HY, 2014 (45) | - | NA | NA | Surg | >6 m (Alive) |
| 51 | Grossman K, 2014 (46) | NA | NA | WT | NA | NA |
| 52 | Al-Najjar H, 2015 (47) | - | NA | WT | Chemo | 12 m (Deceased) |
| 53 | Matsuda Y, 2015 (48) | - | NA | WT | Surg | >12 m (Alive) |
| 54 | Esa NYM, 2018 (49) | - | NA | NA | RT | 7 m (Deceased) |
| 55 | Chen L, 2020 (50) | - | 3% | KRAS mut, TP53 mut, STK11 mut | Chemo + antiangiogenic therapy + IO | 52 m (Deceased) |
| 56 | Chen J-X, 2021 (51) | NA | NA | NA | Untreated | 4 m (Deceased) |
| 57 | Miyama Y, 2020 (52) | - | NA | NA | Chemo + RT | 6 m (Deceased) |
| 58 | Hou Q, 2017 (53) | - | NA | NA | Surg | NA |
| 59 | Zhong MY, 2015 (54) | - | NA | NA | Chemo + RT | NA |
| 60 | He XY, 2020 (55) | - | NA | WT | Chemo | >2 m (Alive) |
| 61 | Pasricha S, 2021 (56) | + | 60% | WT | Chemo | 23 m (Deceased) |
| 62 | Pasricha S, 2021 (56) | + | <1% | WT | RT | 2 m (Deceased) |
| 63 | Pasricha S, 2021 (56) | + | 5% | WT | Chemo | 8 m (Deceased) |
| 64 | Pasricha S, 2021 (56) | + | <1% | WT | Chemo | 0.5 m (Deceased) |
| 65 | Pasricha S, 2021 (56) | + | 80% | WT | Untreated | NA |
| 66 | Pasricha S, 2021 (56) | + | 1% | WT | Chemo | 2 m (Deceased) |
| 67 | Meyers M, 2022 (57) | NA | NA | NA | Chemo + IO + antiangiogenic therapy | 4 m (Deceased) |
| 68 | Yao Y, 2022 (58) | - | NA | WT | Surg | 6 m (Deceased) |
| 69 | Arai H, 2025 (59) | - | 40% | KRAS mut | Chemo + IO + RT | >7 m (Alive) |
| 70 | Cao YA, 2008 (60) | NA | NA | NA | Chemo | >12 m (Alive) |
| 71 | Ding QJ, 2019 (61) | - | NA | WT | Surg + adjuvant Chemo | NA |
| 72 | Xu YJ, 2020 (62) | + | NA | EGFR mut | NA | NA |
| 73 | Xu YJ, 2020 (62) | + | NA | WT | Chemo | 9 m (Deceased) |
| 74 | Fan XL, 2019 (63) | - | NA | NA | Surg | 23 m (Deceased, respiratory failure) |
| 75 | Li Y, 2019 (64) | + | NA | WT | RT | >11 m (Alive) |
| 76 | Yu DZ, 2020 (65) | + | NA | WT | Surg + adjuvant Chemo | 38 m (Deceased) |
| 77 | Shen C, 2021 (66) | - | NA | NA | Surg + adjuvant Chemo | NA |
| 78 | Zeng ZY, 2023 (67) | - | NA | NA | Chemo + antiangiogenic therapy + RT | NA |
| 79 | Cao DM, 2023 (68) | NA | NA | NA | Surg | NA |
| 80 | Cao DM, 2023 (68) | NA | NA | NA | NA | NA |
| 81 | Cao DM, 2023 (68) | NA | NA | NA | NA | NA |
| 82 | Liu GR, 2023 (69) | - | 20% | WT | Chemo + IO | >10 m (Alive) |
| 83 | Zhang K, 2023 (70) | - | NA | NA | Untreated | 6 m (Deceased) |
| 84 | Zhang YY, 2023 (71) | - | NA | WT | NA | NA |
| 85 | Li Q, 2023 (72) | - | 5% | MET amp, TP53 mut | Chemo + RT + targeted therapy | 5 m (Deceased) |
| 86 | Zou LQ, 2023 (73) | - | NA | NA | Surg + adjuvant Chemo + RT | >3 m (Alive) |
| 87 | Zou LQ, 2023 (73) | - | NA | NA | Surg + adjuvant Chemo + RT | >11 m (Alive) |
| 88 | Hong JD, 2025 (74) | - | 5% | TP53 mut, ERCC2 mut | Surg + Chemo + IO + antiangiogenic therapy + RT | >23 m (Alive) |
| 89 | Bai CG, 2006 (75) | NA | NA | NA | Surg | 9 m (Deceased) |
| 90 | Seddon JE, 2021 (76) | + | <1% | NF1 mut, STK11 mut, ARID1B mut, CDKN2A mut, MAGI2 mut, PRDM1 mut, SMARCA4 mut, TP53 mut | Chemo + IO + RT | 17 m (Deceased) |
| 91 | Shahrudin MS, 2023 (77) | NA | NA | NA | Chemo + RT | NA |
| 92 | Cavalcante LB, 2013 (78) | + | NA | NA | Untreated | 1 m (Deceased) |
| 93 | Shao Y, 2016 (79) | NA | NA | WT | Surg + adjuvant Chemo | NA |
| 94 | Lucas C, 2018 (80) | - | NA | NA | BSC | 3 m (Deceased) |
| 95 | Ruiz Cerrato D, 2018 (81) | - | NA | NA | RT | NA |
| 96 | Terracciano LM, 2003 (82) | NA | NA | NA | Surg | 2 m (Deceased) |
| 97 | Genova S, 2003 (83) | NA | NA | NA | Surg | >24 m (Alive) |
| 98 | Kuan K, 2019 (84) | - | NA | WT | Surg | 4 m (Deceased) |
| 99 | Hayashi Y, 2002 (85) | NA | NA | NA | Surg | >32 m (Alive) |
| 100 | No EJ, 2006 (86) | NA | NA | NA | Chemo | >1 m (Alive) |
| 101 | Wang PC, 2008 (87) | NA | NA | NA | Chemo + RT | 14 m (Deceased) |
| 102 | Papatsimpas G, 2012 (88) | - | NA | NA | Chemo + RT | 6 m (Deceased) |
| 103 | Mokrim M, 2012 (89) | - | NA | NA | Chemo | >7 m (Alive) |
| 104 | Che YQ, 2014 (90) | - | NA | NA | Chemo + RT | 20 m (Deceased) |
| 105 | Sun JN, 2016 (91) | - | NA | NA | Surg | >23 m (Alive) |
| 106 | Nakashima K, 2018 (92) | + | NA | NA | Surg | >8 m (Alive) |
| 107 | Shi YF, 2019 (93) | - | <1% | NA | Surg + adjuvant Chemo | 15 m (Deceased) |
| 108 | El Khoury A, 2019 (94) | - | ≥50% | WT | Chemo + IO | >14 m (Alive) |
| 109 | Cancela Díez B, 2019 (95) | NA | NA | WT | Chemo + RT + IO | 7 m (Deceased) |
| **Summary** | | **+:** 31/87 (36%)  **-:** 56/87 (64%)  **NA:** 22 | **<1%:** 9/27 (33%)  **Low expression (1%–49%):** 12/27 (44%) **High expression (≥50%):** 6/27 (22%)  **NA:** 82 | **WT:** 34/53 (64%);  **Genomic alterations detected:** 19/53 (36%);  **Common alterations:** TP53 mut 12/53 (23%), STK11 mut 5/53 (9%), KRAS mut 4/53 (8%), SMARCA4 mut 4/53 (8%), CDKN2A mut 3/53 (6%), EGFR mut 2/53 (4%), MET amp 2/53 (4%); **NA:** 56 | **Treatment information available:** 101/109 (93%)  **Chemo-containing:** 61/101 (60%)  **Surg-containing:** 48/101 (48%)  **RT-containing:** 36/101 (36%)  **IO-containing:** 23/101 (23%)  **Antiangiogenic therapy-containing:** 12/101 (12%)  **Targeted therapy-containing:** 4/101 (4%)  **Untreated/BSC:** 7/101 (7%)  **NA:** 8/109 (7%) | **Deceased:** 49/83 (59%);  **Alive at last follow-up:** 34/83 (41%)**;**  **NA:** 26/109 (24%). |

* **WT**, wild type, negative within the gene-testing scope reported in the original study.

**Abbreviations: amp,** amplification; **BSC**, best supportive care; **Chemo**, chemotherapy; **CN loss**, copy-number loss; **IO**, immunotherapy; **m**, months; **mut**, mutation; **NA**, not available; **OS**, overall survival; **PFS**, progression-free survival; **RT**, radiotherapy; **Surg**, surgery; **WT**, wild type.

** For alive patients, > indicates survival at last follow-up.

Note: Treatment categories were not mutually exclusive, and each modality was counted if it was included in the reported treatment course.
